# HITS-CLIP Analysis Uncovers a Link between the Kaposi’s Sarcoma-Associated Herpesvirus ORF57 Protein and Host Pre-mRNA Metabolism

**DOI:** 10.1371/journal.ppat.1004652

**Published:** 2015-02-24

**Authors:** Emi Sei, Tao Wang, Olga V. Hunter, Yang Xie, Nicholas K. Conrad

**Affiliations:** 1 Department of Microbiology, University of Texas Southwestern Medical Center, Dallas, Texas, United States of America; 2 Department of Clinical Sciences, University of Texas Southwestern Medical Center, Dallas, Texas, United States of America; University of California, Berkeley, UNITED STATES

## Abstract

The Kaposi’s sarcoma associated herpesvirus (KSHV) is an oncogenic virus that causes Kaposi’s sarcoma, primary effusion lymphoma (PEL), and some forms of multicentric Castleman’s disease. The KSHV ORF57 protein is a conserved posttranscriptional regulator of gene expression that is essential for virus replication. ORF57 is multifunctional, but most of its activities are directly linked to its ability to bind RNA. We globally identified virus and host RNAs bound by ORF57 during lytic reactivation in PEL cells using high-throughput sequencing of RNA isolated by cross-linking immunoprecipitation (HITS-CLIP). As expected, ORF57-bound RNA fragments mapped throughout the KSHV genome, including the known ORF57 ligand PAN RNA. In agreement with previously published ChIP results, we observed that ORF57 bound RNAs near the oriLyt regions of the genome. Examination of the host RNA fragments revealed that a subset of the ORF57-bound RNAs was derived from transcript 5´ ends. The position of these 5´-bound fragments correlated closely with the 5´-most exon-intron junction of the pre-mRNA. We selected four candidates (BTG1, EGR1, ZFP36, and TNFSF9) and analyzed their pre-mRNA and mRNA levels during lytic phase. Analysis of both steady-state and newly made RNAs revealed that these candidate ORF57-bound pre-mRNAs persisted for longer periods of time throughout infection than control RNAs, consistent with a role for ORF57 in pre-mRNA metabolism. In addition, exogenous expression of ORF57 was sufficient to increase the pre-mRNA levels and, in one case, the mRNA levels of the putative ORF57 targets. These results demonstrate that ORF57 interacts with specific host pre-mRNAs during lytic reactivation and alters their processing, likely by stabilizing pre-mRNAs. These data suggest that ORF57 is involved in modulating host gene expression in addition to KSHV gene expression during lytic reactivation.

## Author Summary

During viral replication, the oncogenic Kaposi’s sarcoma-associated herpesvirus (KSHV) modulates both host and viral gene expression. KSHV ORF57 is a multifunctional posttranscriptional regulator that is essential for viral replication and stabilizes viral RNAs. Previous studies demonstrated that ORF57 RNA-binding is essential for its activity, but the full spectrum of ORF57 targets are unknown. Here we employed a high-throughput analysis to identify RNA fragments bound by ORF57 during lytic reactivation. As expected, we found targets that mapped to the viral genome, and we further uncovered novel host targets, a subset of which had ORF57 bound near their 5´ ends. Further examination of this subset demonstrated that ORF57 bound preferentially at the 5´-most exon-intron boundary. ORF57 affected the pre-mRNA abundance from these genes, most likely by stabilizing otherwise unstable inefficiently spliced pre-mRNAs. In at least one case, this stabilization led to increases in mRNA expression of the host gene. We suggest that KSHV employs the same mechanism to stabilize intronless viral RNAs and cellular unspliced pre-mRNAs to modulate viral and host gene expression during lytic reactivation.

## Introduction

Kaposi’s sarcoma-associated herpesvirus (KSHV; HHV-8) is a human gammaherpesvirus and the etiological agent for Kaposi’s sarcoma (KS), primary effusion lymphoma (PEL), KSHV-inflammatory cytokine syndrome (KICS), and some cases of multicentric Castleman’s disease (MCD)[1–4]. The KSHV life cycle includes both a latent and a lytic state, which require different viral gene expression programs and distinct interactions with the infected host cell [5–9]. During latency, a small subset of KSHV genes is expressed that allows propagation and maintenance of the KSHV genome in the absence of viral replication. In contrast, during lytic reactivation KSHV orchestrates the ordered synthesis of numerous viral products that enable assembly of viral particles. The timing and amount of expression for each gene product is important for efficient production of infectious virions. During both lytic reactivation and latency, the virus manipulates the cell environment and gene expression machinery to modulate human and viral gene expression.

One KSHV factor critical for viral gene expression is the ORF57 protein (Mta) [10–12]. While no host homologs are known, every herpesvirus encodes a homolog of ORF57 and each is essential for virus replication [13–15]. ORF57 is multifunctional, but most of its known activities are associated with posttranscriptional regulation of gene expression. For example, ORF57 has been reported to increase the export of intronless viral RNAs by interaction with the cellular REF/Aly protein, or the related UIF protein [16–18]. In this model, ORF57 serves as a bridge between the viral RNAs and the cellular transcription and export (TREX) complex, a multisubunit protein complex including the REF/Aly or UIF proteins that promotes cellular mRNA export [19,20]. TREX is recruited to the 5´-end of cellular RNAs as a result of splicing [21], but this mechanism is not feasible for the virus since most KSHV genes are intronless [22]. Therefore, it is a compelling hypothesis that ORF57 compensates for the lack of splicing of intronless genes by recruiting the TREX complex to unspliced viral genes. Indeed, ORF57 enhances the RNA expression of various intronless reporters [18,23–34], but has little effect on analogous intron-containing genes [27,29,33]. It is important to note that the proposed role of ORF57 in mRNA export remains somewhat controversial. In some cases, little or no effect on mRNA nucleocytoplasmic RNA distribution has been observed with intronless reporters [23,25,31,35]. In addition, point mutations that abrogate the ORF57 interaction with REF/Aly support viral replication [36].

ORF57 stabilizes viral RNAs in the cell nucleus, independent of its reported ability to export intronless RNAs. This function was first suggested by the observation that the levels of the polyadenylated nuclear (PAN) RNA is up-regulated by co-expression of ORF57 in transient transfections[31,33]. Direct determination of PAN RNA half-lives further showed increases in PAN RNA levels upon co-expression of ORF57 [27,37]. Moreover, PAN RNA levels are reduced in cells infected with an ORF57-null bacmid [38,39]. In addition to PAN RNA, ORF57 increases the nuclear and cytoplasmic abundance of specific viral mRNAs [23,25,31,35]. Presumably, the protection of these RNAs by ORF57 in the nucleus ultimately leads to more viral intronless RNAs escaping nuclear decay and being transported to the cytoplasm. In fact, intronless transcripts are subject to a polyadenylate (poly(A))-tail dependent nuclear RNA decay pathway [40,41]. Therefore, the apparent specificity of ORF57 for intronless RNAs may be dictated by their susceptibility to this RNA decay pathway, but this has yet to be formally tested. Of course, functions for ORF57 in nuclear RNA stability and mRNA export are not mutually exclusive. In addition, ORF57 promotes viral RNA splicing [42], regulation of host gene expression [43], genome instability [44], translation [45], and may be involved in transcription [33,34,46,47].

ORF57 binds directly to RNA, but whether binding is driven by specific RNA sequences, cellular factors, and/or if ORF57 binding is coupled to RNA synthesis or processing remains unclear. In the case of PAN RNA, two groups reported the presence of specific sequences in the 5´ end of PAN RNA, dubbed the ORF57-responsive element (ORE) [28] or Mta-responsive element (MRE)[26]. The PAN RNA ORE was necessary and sufficient for maximal ORF57-responsiveness of PAN RNA and heterologous reporters. An additional MRE was found in the viral IL6 transcript, further supporting the idea that ORF57 binding is driven by specific sequences [43]. However, ORF57 is capable of robust enhancement of the levels of artificial intronless reporters that did not co-evolve as ORF57 ligands (e.g. β-globin, CAT, luciferase), suggesting that its binding may be nonspecific or driven by binding of general cellular factors [27–29,33,35,48]. Importantly, RNA binding is important for ORF57 function [18,24,27,31]. When the ORE was deleted from PAN RNA, ORF57 had a significantly reduced effect on PAN RNA levels. However, this could be complemented by artificially tethering ORF57 to PAN RNA, formally demonstrating that association of ORF57 to PAN RNA was necessary and sufficient for PAN RNA up-regulation [27].

Because of the importance of ORF57 RNA-binding for function, a comprehensive understanding of the RNAs bound by ORF57 during lytic infection is required to understand ORF57’s essential activities in viral replication. Indeed, an unbiased screen for ORF57-bound RNAs revealed novel targets, including one host RNA (IL6 mRNA), and suggested a new ORF57 mechanism [43]. However, this study was low throughput compared to the next generation sequencing methodologies currently available. Global analysis of RNA-binding proteins is complicated by the propensity for RNA-binding proteins to reassort in cell extract [49,50]. That is, RNA-binding proteins will potentially lose or gain interactions with RNAs that are not bound in cells, so this caveat must be accounted for in data collection and interpretation. Our previous studies demonstrated that ORF57 binds targets in extract upon cell lysis that were not bound in vivo [27], thereby necessitating the use of crosslinking methods to analyze ORF57-RNA interactions in cells.

In this study, we have adapted high-throughput sequencing of RNA isolated by crosslinking immunoprecipitation (HITS-CLIP) for identification of ORF57 targets during lytic reactivation [51]. As predicted, we identified CLIP tags mapping to the 5´ end of PAN RNA and additionally observed ORF57 interactions with RNAs generated at the KSHV origins of lytic replication (oriLyt). Examination of host targets revealed ORF57 binding sites near the 5´ end of a subset of the transcripts and these often mapped close to the first exon-intron junction. We then monitored the RNA levels of four potential ORF57 targets (BTG1, EGR1, TNFSF9, and ZFP36) at various times following lytic induction. Interestingly, the levels of these ORF57-bound pre-mRNAs persisted longer than controls, suggesting that ORF57 may be stabilizing these cellular pre-mRNAs. Using a metabolic labeling strategy, we selectively monitored transcripts synthesized over a short time window subsequent to viral reactivation. Consistent with the steady-state experiments, we observed that pre-mRNA levels of the ORF57 targets were higher after induction relative to controls that were not bound by ORF57. Most importantly, we show that ORF57 is sufficient to increase the pre-mRNA levels of BTG1, EGR1, and ZFP36, but its effects on mRNA levels differ. EGR1 mRNA abundance was increased by ORF57 while BTG1 and ZFP36 mRNA levels were largely unaffected. We suggest these transcript-specific distinctions are due to cell-type specific differences in the relative splicing and decay efficiencies of these RNAs in the nucleus. These studies represent the first high-throughput analysis of ORF57-bound RNAs and they provide insights into ORF57 interactions with host and viral RNAs during lytic reactivation.

## Results

### Identification of ORF57-bound RNAs by HITS-CLIP

To identify RNAs bound by ORF57 during lytic reactivation, we performed HITS-CLIP [51,52] in lytically reactivated TREx BCBL1-Rta cells [53]. In these cells, the gene encoding the KSHV transcription factor Rta is under the control of a tetracycline/doxycycline-inducible promoter. Rta expression is necessary and sufficient to drive KSHV lytic reactivation. We used both doxycycline and the histone deacetylase inhibitor sodium butyrate to achieve the highest levels of reactivation. HITS-CLIP analysis controls for reassortment of RNAs and proteins in extract by employing ultraviolet (UV) irradiation to covalently crosslink proteins to their ligand RNAs. Extracts are then generated, nuclease treated to partially digest RNA, and immunoprecipitations are performed under high stringency conditions. The immunoprecipitated RNA fragments are radiolabeled allowing them to be visualized after they are run on a denaturing PAGE and transferred to a nitrocellulose membrane. Protein-RNA complexes are cut from the nitrocellulose membrane, treated with protease, and libraries made from the resulting immunoprecipitated and gel-purified RNAs are analyzed by high-throughput sequencing.

**Fig 1:**
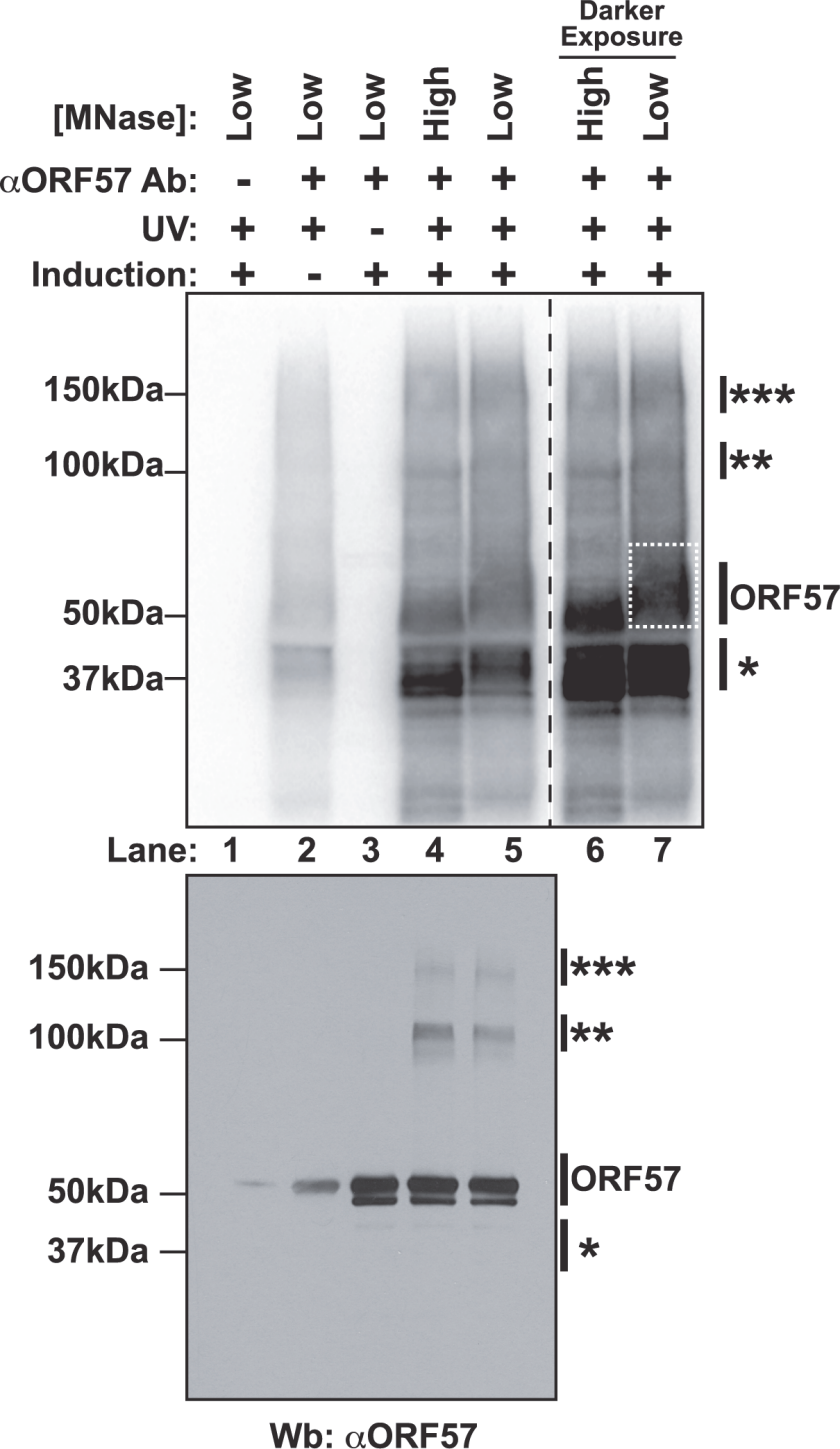
Isolation of cross-linked ORF57-RNA complexes for HITS-CLIP analysis. *Top* Nitrocellulose membrane from an ORF57 immunoprecipitation performed under HITS-CLIP conditions. Cross-linked RNA fragments were end-labeled with ^32^P to be visualized by Phosphoimager. Cells were induced to undergo lytic reactivation, exposed to UV and/or treated with high or low concentrations of MNase as indicated. The samples lacking an ORF57 antibody (lane 1) were precipitated with pre-bleed antibodies from the same rabbit. Lanes 6 and 7 are a dark exposure of lanes 4 and 5. The dashed white box indicates the position of the ORF57 complex cut from the membrane for library preparation. *Bottom* Western blot of the same HITS-CLIP samples shown in the top panel. Affinity purified rabbit anti-ORF57 was used to detect ORF57. Positions of molecular weight markers are shown on the left. On the right, a single asterisk marks the position of a contaminating ~37 kDa protein; double and triple asterisks mark positions of putative ORF57 homodimers and homotrimers bound to the same RNA. The doublet is likely due to an ORF57 cleavage product [104].

Due to ORF57’s propensity to be lost in insoluble nuclear fractions and to precipitate in lysate, we had to perform extensive optimization of the cross-linking and extract preparation steps. Under these conditions, immunoprecipitation of RNA was undetectable when we immunoprecipitated with non-specific antibodies (Fig. 1, lane 1, top). When the cells were not exposed to UV light, no RNA was present (lane 3, top), but the protein remained efficiently precipitated as determined by western blotting of the immunoprecipitated complexes (lane 3, bottom). Uninduced samples retain a weak signal at both the RNA and protein levels (lane 2), presumably due to a small population of cells undergoing spontaneous lytic reactivation [54]. Upon treatment with high concentrations of micrococcal nuclease (MNase), we observed several bands. One protein-RNA complex migrated near 50 kDa (lanes 4 and 6), consistent with the 51 kDa ORF57, and this band shifts to a higher molecular weight (MW) smear when the MNase concentration was decreased (lanes 5 and 7). In addition to this 50-kDa complex, we observed signals from a lower MW complex (~37kDa, single asterisk) and fainter bands migrating at ~100 kDa and ~150 kDa (double and triple asterisks, respectively). Western blotting confirmed that the 50-kDa, 100-kDa, and 150-kDA bands correspond to ORF57, whereas the 37-kDa band did not react with ORF57 antibodies (lanes 4 and 5, bottom). The 100-kDa and 150-kDa ORF57 protein bands are only present upon treatment with UV light. We interpret these data to represent complexes in which two (100 kDa) or three (150 kDa) ORF57 protein molecules were cross-linked to the same RNA. We think this is a reasonable interpretation given the sizes of the complexes, the previous demonstration of ORF57 homomultimerization [31,55], and a similar phenomenon was observed in HITS-CLIP studies of the TDP-43 RNA binding protein [56].

We further characterized the cross-linked immunoprecipitated complexes by analyzing the RNA in the complexes. First, we examined the length of the RNAs in each protein-RNA complex and found that RNA purified from the higher molecular weight complexes had a longer average size than the lower molecular weight complexes (S1A Fig.). This observation further supports our interpretation that the slower migrating complexes are from ORF57 multimers bound to the same RNA, as this would require a longer RNA platform for multimerization. Second, we rationalized that we could use the presence of PAN RNA in the different MW complexes as a measure of biologically relevant ORF57-RNA complexes. We analyzed RNAs purified from the different MW complexes by northern blots. PAN RNA was immunoprecipitated from the putative ORF57 complexes, but was largely absent from the 37-kDa complex (S1B Fig.). In contrast, when we probed for the 5.8S rRNA, a common contaminant due to the high abundance of rRNA, the signal was stronger in RNAs derived from the 37-kDa band (S1C Fig.). Thus, we conclude that both the 50-kDa, 100-kDa, and 150-kDa RNA-protein complexes contain ORF57 and its bound RNAs, whereas the 37-kDa band is a contaminating protein. Therefore, we made libraries of RNA fragments derived from the 50-kDa complex (Fig. 1A, dashed box, lane 7) and sequenced them using strand-specific high throughput sequencing methods. Herein we refer to the reads from this library as the “CLIP tags” or “pellet” samples. As reference samples, we generated libraries from RNA isolated from the induced cells immediately prior to UV treatment; these libraries are referred to as “input” samples.

### Bioinformatic analysis of HITS-CLIP data

**Fig 2:**
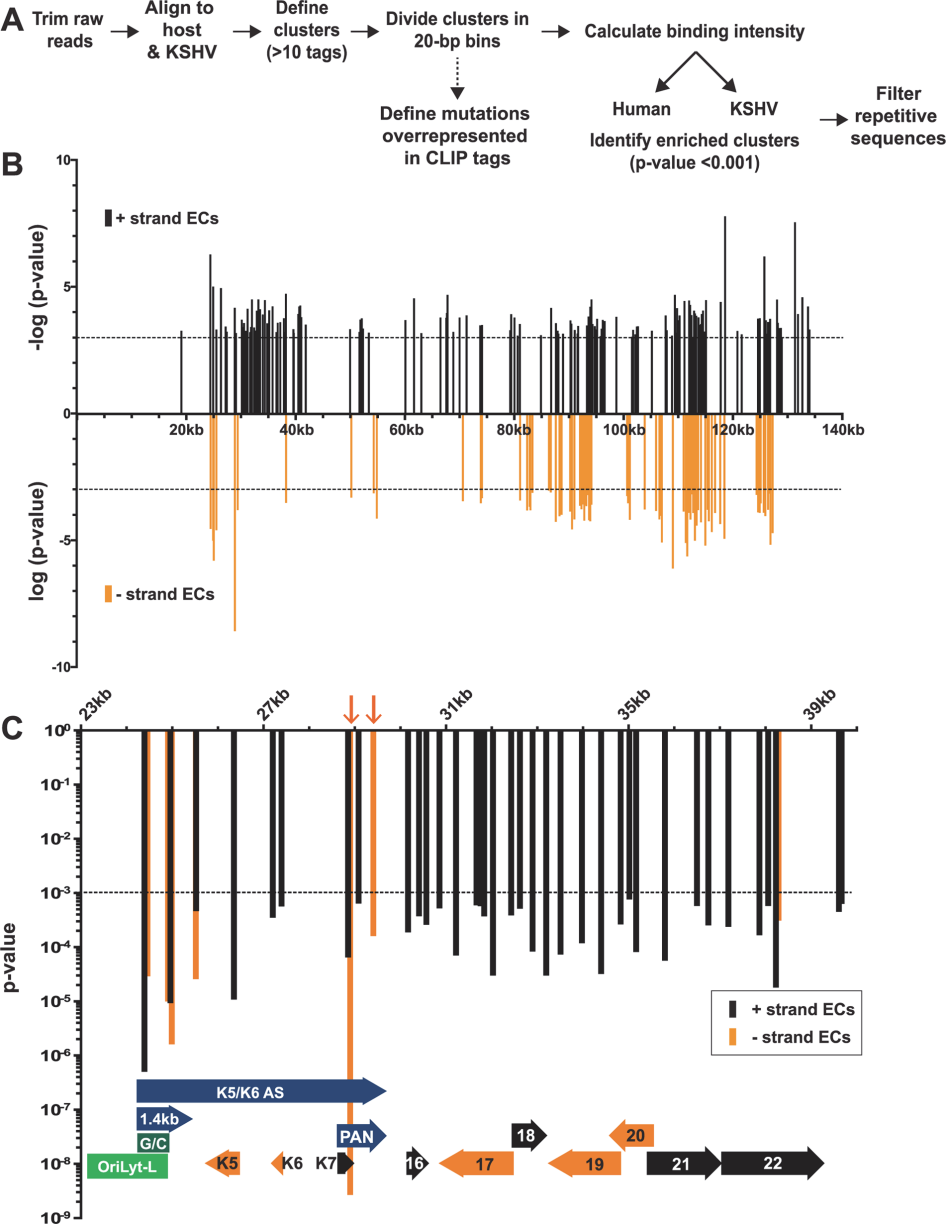
Identification of enriched clusters mapping to the KSHV genome. (A) Outline of the bioinformatic pipeline used to identify enriched clusters. (B) Genomic location of KSHV enriched clusters. The x-axis represents position on the KSHV genome (U75698) and the midpoint of each cluster was used as the x-coordinate. KSHV enriched clusters ranged from 19–3679 nt; the mean enriched cluster length was 176 nt and the median was 79 nt. A statistical cutoff of 0.001 was used to define enriched clusters (dashed lines). For display, the RNA fragments mapping to the KSHV plus strand were assigned -log_10_(p-values) (black) while the minus strand clusters are displayed as log_10_(p-values) (orange). (C) Detailed examination of the enriched clusters from KSHV genome position 23kb-40kb. The plus and minus strands are in black and orange as in (B) but both are displayed as p-value. For the genome annotations shown at the bottom of the graph, plus strand ORFs are black arrows, minus strand ORFs are orange arrows, oriLyt-L elements are in green, and RNAs with potential noncoding functions are purple. Orange arrows point to enriched clusters on PAN RNA minus strand.

We developed a pipeline for HITS-CLIP analysis to identify continuous regions of the genome where CLIP tags were enriched when compared to the equivalent region in the input samples. We dubbed these regions enriched clusters and they represent RNA fragments bound by ORF57. The general workflow for enriched cluster identification is given in Fig. 2A and further details are provided in Materials and Methods. After trimming, raw reads were simultaneously aligned to the KSHV and human genomes. Any group of >10 overlapping tags was defined as a cluster and each cluster was divided into 20-bp bins. The covalent crosslinking of proteins to RNA often generates mutations during reverse transcription due to cross-linked protein adducts remaining on the RNA [57]. In addition, protein-RNA crosslinks generate types of mutations that are characteristic to the specific protein being crosslinked. The presence of such mutations within a putative binding site increases confidence that the identified CLIP tags are bona fide binding sites. We determined whether any classes of mutations were overrepresented in the CLIP tag clusters compared to the input clusters. Using various criteria, we defined T→C transitions and nucleotide deletions to be characteristic to ORF57 crosslinking (see Materials and Methods). Further bioinformatic analyses resulted in the assignment of a p-value to the CLIP tag bins and a statistical cutoff of p-value<0.001 was applied to each bin; adjacent bins reaching this threshold were combined to defined enriched clusters. The p-value assigned to the enriched cluster is the average p-value of the bins that constitute that enriched cluster. Most importantly, this p-value encompasses three parameters. First, the CLIP tag clusters were referenced to the input clusters to obtain a fold enrichment value. A high fold enrichment value denotes that the number of tags in a specific pellet cluster relative to the corresponding input cluster was higher than this pellet-to-input ratio for the entire dataset. Clusters with larger fold enrichment values were given increased statistical significance. Second, the presence of T→C transitions or nucleotide deletions in the CLIP tag clusters provided additional statistical weight, but not all enriched clusters contain mutations. Third, the reproducibility of the data among the three biological replicates was considered for the clusters. In general, we observed high reproducibility among our three biological replicates across both the input and pellet samples (S2 Fig.). These analyses led to the identification of 2,448 enriched clusters mapping to both the KSHV (219) and human (2,229) genomes (S1 Table). We additionally applied a looser statistical cutoff to our data (p<0.05) because at least one known ORF57 target was not recovered in the higher stringency data set (see below). This analysis led to 6,933 and 343 enriched clusters mapping to the human and viral genomes, respectively (S1 Table). The analysis detailed here refers to the high stringency data set (p<0.001) unless otherwise noted.

### HITS-CLIP reveals ORF57-RNA interactions in the KSHV transcriptome

**Fig 3:**
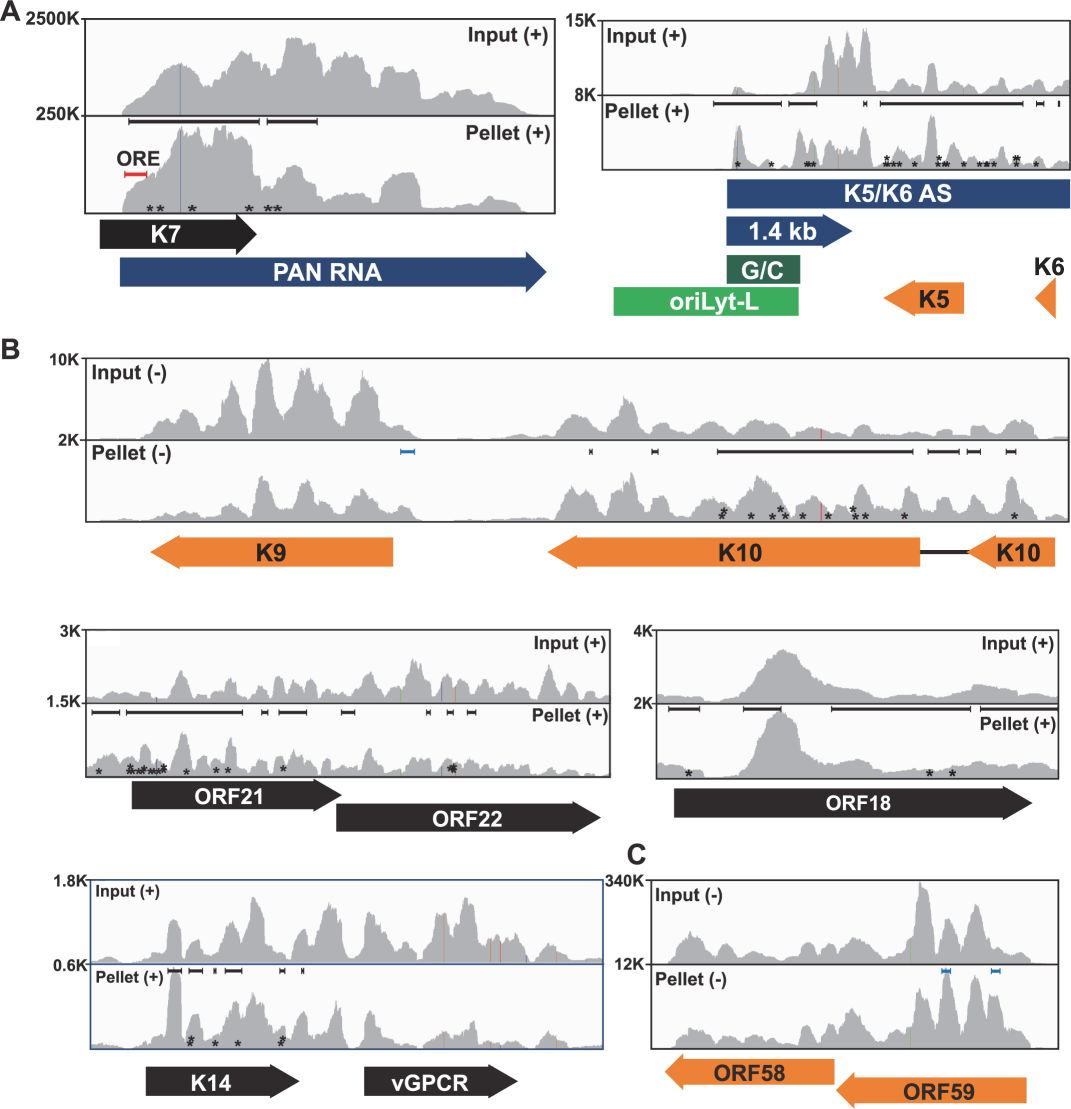
Sequence reads across various viral genomic loci. Mapped sequence reads of input (top) and pellet (bottom) from one biological replicate for the region surrounding (A) PAN RNA (left) and oriLyt-L (right). (B) Additional loci in which enriched clusters were observed in the high stringency dataset. (C) ORF58-ORF59 region, which was only identified in the low stringency dataset. In all panels, reads from only one strand are shown which is indicated by the plus or minus signs. The black bars above the peaks in the pellet panels mark the positions of the enriched clusters from the high stringency dataset whereas the blue bars were observed only in the low stringency dataset. The color schemes for genes are the same as Fig. 2. The numbers at the top left of each frame are proportional to the number of reads stacked in a given window. The asterisks approximate the positions of T→C or deletion mutations observed in the pellets.

Our analysis identified 219 enriched clusters in the viral genome. The enriched clusters mapped broadly across the KSHV genome and were observed on plus and minus strand RNAs, consistent with a general role for ORF57 in KSHV RNA biogenesis (Fig. 2B). As expected, we identified enriched clusters in PAN RNA and the identified clusters were located near the 5´ end of the RNA (Fig. 3A) [26–28]. Interestingly, induced mutations were observed at several positions across the transcript, suggesting multiple binding sites of ORF57 on the RNA (Fig. 3A, asterisks), which likely result from ORF57 multimerizing along the transcript. Surprisingly, we observed two enriched clusters mapping to the minus strand along PAN RNA (Fig. 2C, orange arrows). To our knowledge, no transcripts have been identified in this orientation from this location [7] and we observed that some of the other enriched clusters were on the opposite strand from known transcripts. We can think of three possible explanations for this observation. First, this could be an artifact in library preparation. Given the excessively high abundance of PAN RNA [7,58], errors in preserving strand specificity in a small fraction of the RNAs could lead to observable peaks. Second, these data could be pointing to novel transcripts. However, we saw no discrete bands that were induced upon virus induction using a PAN RNA sense strand as a probe and deep RNA-seq studies did not identify a transcript at this locus [7], so we think this is unlikely. Third, given the high transcription rates of KSHV genome during viral replication, we can imagine that some transcriptional noise occurs across the genome. If ORF57 binds to RNAs co-transcriptionally, the resulting transcripts may bind to ORF57. In any case, the identification of enriched clusters at the 5´ end of PAN RNA demonstrates that our HITS-CLIP analysis successfully identified ORF57-bound RNA fragments.

KSHV has two origins of lytic replication called oriLyt-L and oriLyt-R that contain AT-rich palindromes and GC-rich regions [59–61]. The oriLyt-L region is examined more closely in Figs. 2C and 3A (green). The 1.4-kb transcript produced from oriLyt-L is essential for replication (1.4 kb, purple). The function of the 1.4 kb RNA is not clear, but it contains a small ORF and could serve additional functions as a noncoding RNA [7,62,63]. Two enriched clusters overlapped with the 1.4-kb RNA: one spanned the GC-rich region while the second was 3´ to the GC-rich region (Fig. 3A). The ORF57 interaction with RNA at oriLyt-L extends observations from chromatin immunoprecipitation (ChIP) studies that demonstrated an association between ORF57 and oriLyt-L DNA [46]. The observed ChIP peak approximately corresponds to the position of the 1.4-kb RNA and the identification of enriched clusters at the oriLyt suggests that the interaction with viral DNA observed in the ChIP assays was due to ORF57 binding to the 1.4 kb RNAs. However, based on the HITS-CLIP data alone, we cannot distinguish whether the association between ORF57 and RNA in this region is due to an interaction with the 1.4 kb RNA or with the overlapping K5/K6 antisense transcript [7,62] (Fig. 3A). Indeed, we found four enriched clusters that unambiguously map to the K5/K6 antisense transcript, so ORF57 could be solely binding this RNA or to both the 1.4-kb RNA and the K5/K6 antisense RNA. Based on the ChIP data, we suggest that the latter is more likely.

We show other viral enriched clusters mapping to the K10, ORF18, and bicistronic ORF21-ORF22 and K14-vGPCR loci (Fig. 3B). We included both the K10 and K9 genes for easy comparison of the relative levels of input and pellet samples between a transcript with enriched clusters and one without enriched clusters. We were surprised that we identified no enriched clusters mapping to the ORF59 mRNA (Fig. 3C), a well-characterized target of ORF57-mediated up-regulation [23–25,30,31,38,45]. Visual inspection of the ORF59-ORF58 locus showed a peak near the 5´ end of ORF59 that is more prevalent in the pellet than input samples, consistent with an ORF57-binding site, but the fold enrichment for this peak did not reach the levels of significance used as our cutoff (p<0.001). Of course, statistical cutoffs are necessarily arbitrary and this example emphasizes that the statistical cutoffs we originally used to define enriched clusters were conservative. This prompted us to repeat the analysis with a less stringent statistical threshold (p<0.05). As expected, we see more enriched clusters across the viral genome (S3 Fig.) and enriched clusters were identified in ORF59 and in K9 (Fig. 3B and 3C, blue bars). Given the extent of binding observed, our data are consistent with a relatively general mode of binding by ORF57 to KSHV RNAs. However, in this study, we will shift our focus to novel ORF57-bound host (pre-)mRNAs.

### ORF57 binds the 5´ ends of a subset of host (pre-)mRNAs

**Fig 4:**
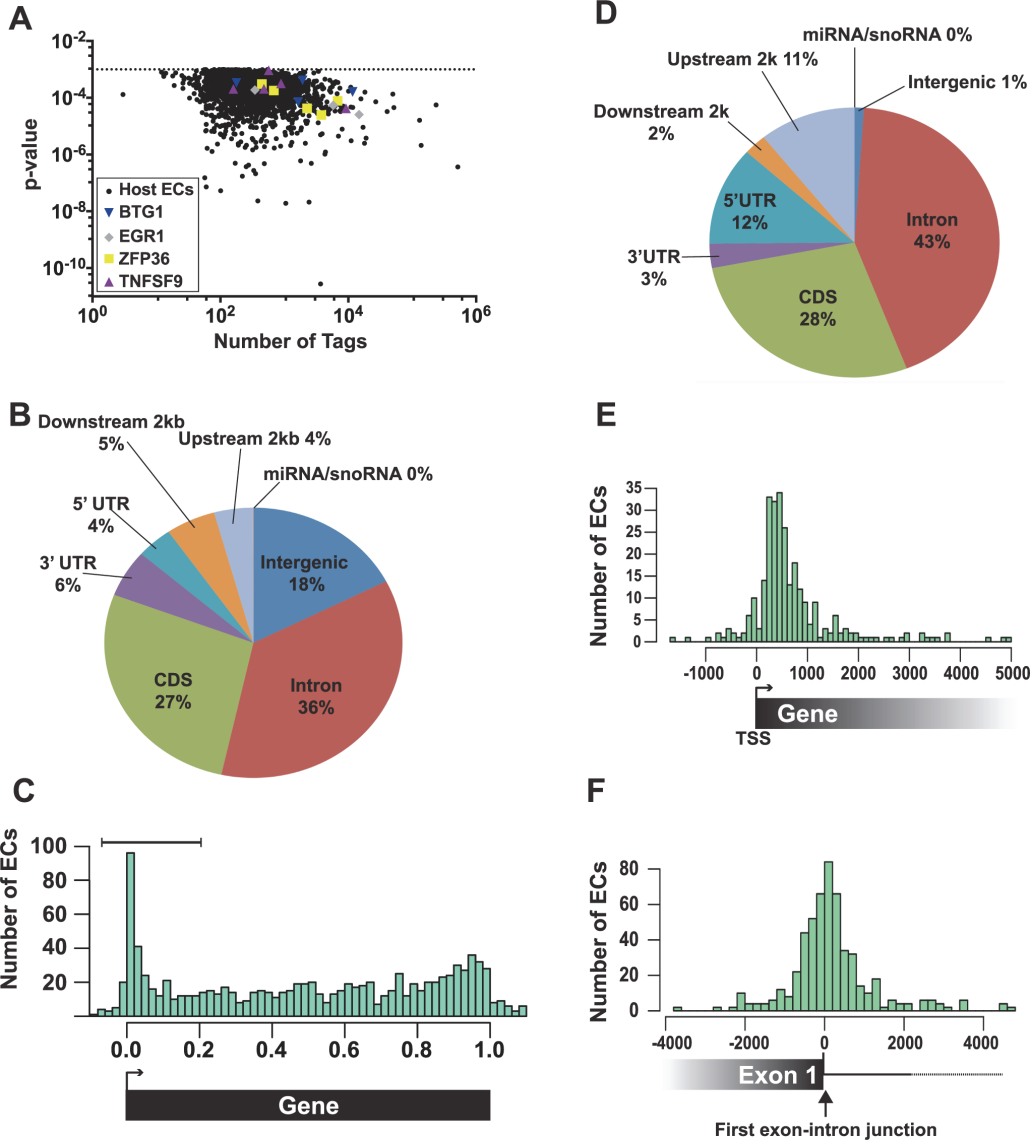
Identification and characterization of enriched clusters mapping to the human genome. (A) Graph of the 2,229 host enriched clusters with their p-values plotted on the y-axis and the total tag count for one of the three biological replicates plotted on the x-axis. Enriched clusters from four selected candidate genes are shown in color. (B) Pie chart of the gene feature annotations of human enriched clusters. If a cluster spanned multiple different annotations, the cluster annotation was split proportionally among the annotations. Upstream and downstream 2 kb refer to clusters mapping within 2 kb immediately flanking the nearest annotated gene. (C) The bar graph compares the number human enriched clusters (ECs) relative to their position on the closest annotated transcript. 0.0 and 1.0 correspond to the transcription start site (TSS) and the poly(A) site, respectively. Importantly, the length in this graph is relative to the annotated gene and is not a measure of the distance in base pairs. The brackets denote the 5´-enriched clusters examined in D-E. (D) Pie graph classifies the 5´-enriched clusters based on their gene feature annotations (n = 288). (E) Bar graph comparing the number (y-axis) and position (x-axis) of 5´ enriched clusters relative to the transcription start site (TSS). The distance is measured in base pairs from the TSS (TSS = 0). (F) Bar graph comparing the number (y-axis) and position (x-axis) of 5´ enriched clusters relative to the 5´-most exon-intron junction. The distance is measured in base pairs from the first exon-intron boundary, which was set at zero.

A total of 2,229 enriched clusters mapped to the human genome and these ORF57-bound RNA fragments correspond to ~700 unique host genes. The human enriched clusters were derived from clusters with tag counts spanning several orders of magnitude, so we can be confident that our bioinformatics pipeline has a wide dynamic range (Fig. 4A). We determined where the enriched clusters mapped in relationship with specific gene features. Over one third of the clusters mapped to introns, while 27% was found in coding sequences (Fig. 4B). To look for biases in the location of enriched clusters across genes, we calculated where each enriched cluster midpoint maps as a fraction of the length of that specific gene. The results were subsequently compiled to a single model gene (Fig. 4C). While enriched clusters were found throughout the length of target genes, we observed a clear overrepresentation of enriched clusters near the 5´ ends of genes (Fig. 4C). The enriched clusters concentrated at 5´ ends (Fig. 4C, bracket) were examined based on where they mapped to gene features (Fig. 4D). As expected for 5´-enriched fragments, we detected increases in the percentage of enriched clusters mapping to the 5´ UTR and upstream 2 kb and decreases in the intergenic regions, downstream 2 kb and 3´ UTR annotations. We further observed a small increase in the percent mapping to intronic regions (36% in the total and 43% in the 5´-most clusters), but it is unclear whether this increase is significant. Next, we determined the distances between the transcription start sites (TSS) and the 5´ enriched clusters and observed that the 5´ enriched clusters do not peak directly at the TSS, but rather ~300–500 bp downstream of the TSS (Fig. 4E). In contrast, when we examined the distances between the 5´ enriched clusters and the first exon-intron boundary, we observed a peak coincident with this boundary (Fig. 4F). Consistent with the observed 43% intronic reads, the peak is not solely on the exonic sequence but spans the exon-intron junction. These characteristics were nearly identical for the low stringency dataset (S4 Fig.), suggesting that this dataset identified additional ORF57-bound RNAs. Taken together, these data show that a subset of the ORF57-bound RNA fragments map to the 5´ end of the transcript and are particularly concentrated near the 5´-most exon-intron boundary.

The enriched clusters were found in RNAs encoding proteins involved in a wide variety of functions including RNA processing, DNA metabolic processes, and cell cycle processes (S2 Table). In particular, RNA processing was enriched in the 5´-enriched, high and low stringency, which is interesting given ORF57s functions in posttranscriptional gene regulation. We did not identify enriched clusters in human IL6 mRNA [43], but the IL6 RNA levels were low in the input samples, so this result is inconclusive. To validate our HITS-CLIP assay, we chose four candidate transcripts from the 5´ enriched clusters data set for further analysis: EGR1, ZFP36, BTG1, and TNFSF9 (Fig. 4A, colored shapes). We selected these RNAs based on their expression levels, fold enrichment values (S1 Table), and their potential biological relevance to KSHV pathogenesis. EGR1 (ZNF225, KROX24, AT225) is a highly regulated putative tumor suppressor gene that regulates the transcription of genes involved in multiple cellular processes such as inflammation and apoptosis [64,65]. ZFP36 (TTP/tristetraproline, GOS24, TIS11A) regulates cell proliferation and inflammation by binding 3´ UTRs with AU-rich RNA elements and regulating mRNA stability [66]. BTG1 is a transcription factor that has also been implicated in cytoplasmic mRNA decay. BTG1 negatively regulates cell proliferation and BTG1 mutations are associated with leukemias [67,68]. TNFSF9 (CD137L, 4–1BBL) is involved in T-cell activation, but its expression is associated with B-cell lymphomas [69–71]. These genes all function to modulate cell growth and therefore their regulation has potential relevance to KSHV pathogenesis and/or life cycle.

**Fig 5:**
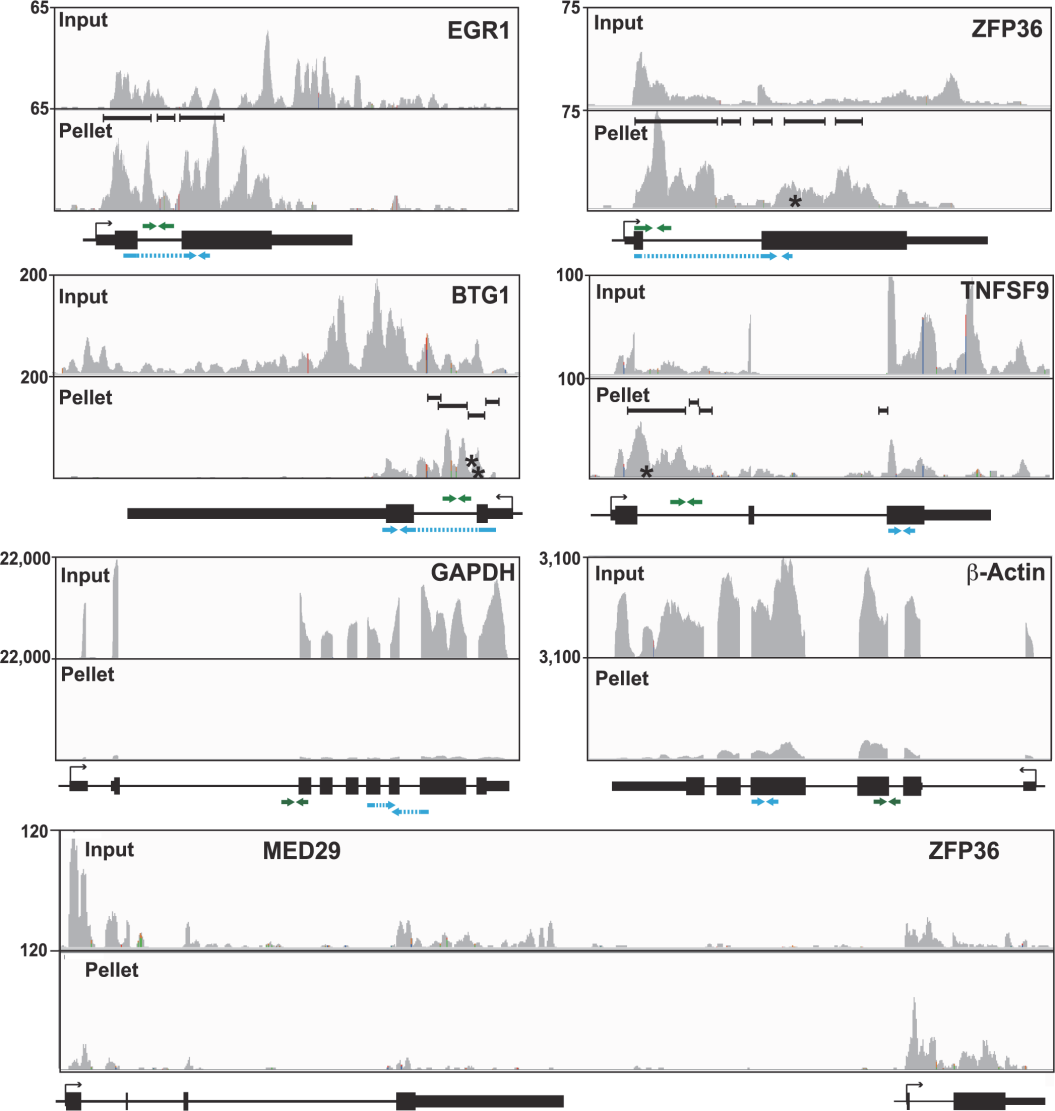
Sequence traces of ORF57-bound human RNAs. Each panel is a screen shot (IGV browser [105]) of the input and pellet samples from one biological replicate. The numbers at the top left of each frame are proportional to the number of reads stacked in a given window. The x-axes in each panel were scaled for each specific transcript. The black bars in the pellet panels denote positions of the enriched clusters and the asterisks denote approximate positions of characteristic mutations. Below each panel is a gene diagram with the TSS and gene orientation indicated by the black arrow. Green and blue arrows above and below the gene diagram indicate the positions of primers used to assay pre-mRNA and mRNAs, respectively. The exons (thick black rectangles) and introns (black lines) are to scale, but the primer and amplicon lengths are not. When possible, primers or amplicons for mRNAs were designed to span exon-exon boundaries (blue dashed lines).

Inspection of the sequence traces confirmed the presence of enriched clusters mapping toward the 5´ ends of these RNAs but not in the GAPDH or β-actin controls (Fig. 5). Multiple enriched clusters were found in each of these RNAs: three, five, four, and four enriched clusters were identified for EGR1, ZFP36, BTG1, and TNFSF9, respectively (Fig. 5, black bars above pellet reads). These data are similar to what was observed with PAN RNA (Fig. 3A) and consistent with the proposal that once ORF57 binds an RNA, it subsequently binds several places along the transcript due to its multimerization [28]. In fact, nearly half of the genes identified in the high stringency dataset (44.7%) and over half of the genes (58.7%) in the low stringency dataset had more than one enriched cluster (S5 Fig.). Moreover, the enriched clusters in introns are readily observable in the sequence traces (Fig. 5). As expected, the proportion of HITS-CLIP tags relative to the input samples is considerably lower for GAPDH and β-Actin as these RNAs contained no enriched clusters. The MED29 gene contains no enriched clusters, but is located immediately upstream of ZFP36 and is transcribed from the same strand as ZFP36. We included the sequence traces for the MED29-ZFP36 locus, because visualization of both of these genes provides a convincing internal control for the identification of enriched clusters (Fig. 5, bottom). In this case, the input levels of MED29 RNA were similar to or even slightly higher than those for ZFP36. In contrast, the number of tags found in the pellet samples was overrepresented in the ZFP36 samples when compared to MED29, consistent with our identification scheme for enriched clusters. Overall, this visual inspection supports the conclusion that our bioinformatic pipeline is reliable and identified ORF57-bound RNA fragments within introns and 5´ ends of host genes. Moreover, these results suggest that the four transcripts are reasonable candidates to further investigate as potential functional targets of ORF57 activity.

Interestingly, BTG1, EGR1, and ZFP36 genes all contain only a single intron and TNFSF9 has two introns (Fig. 5) whereas the average intron number in humans is ~7–8 [72]. Moreover, ORF57 upregulates intronless transcripts [11–13,73], so we asked whether genes with few introns were overrepresented among the host RNAs we identified. However, this was not the case (S5 Fig.). The average number of exons in the annotated genes represented by all of our enriched clusters, was not significantly lower than the exon number in the entire genome. Similarly, the subset of genes with 5´-end enriched clusters does not deviate considerably from the average number of exons compared to the entire genome. There is, however, a shift in the average exon number of clusters enriched at the 3´ ends of transcripts towards a larger number of exons, but it is not clear whether this is biologically relevant.

### Candidate ORF57 target pre-mRNAs persist longer than control pre-mRNAs over the course of KSHV lytic reactivation

**Fig 6:**
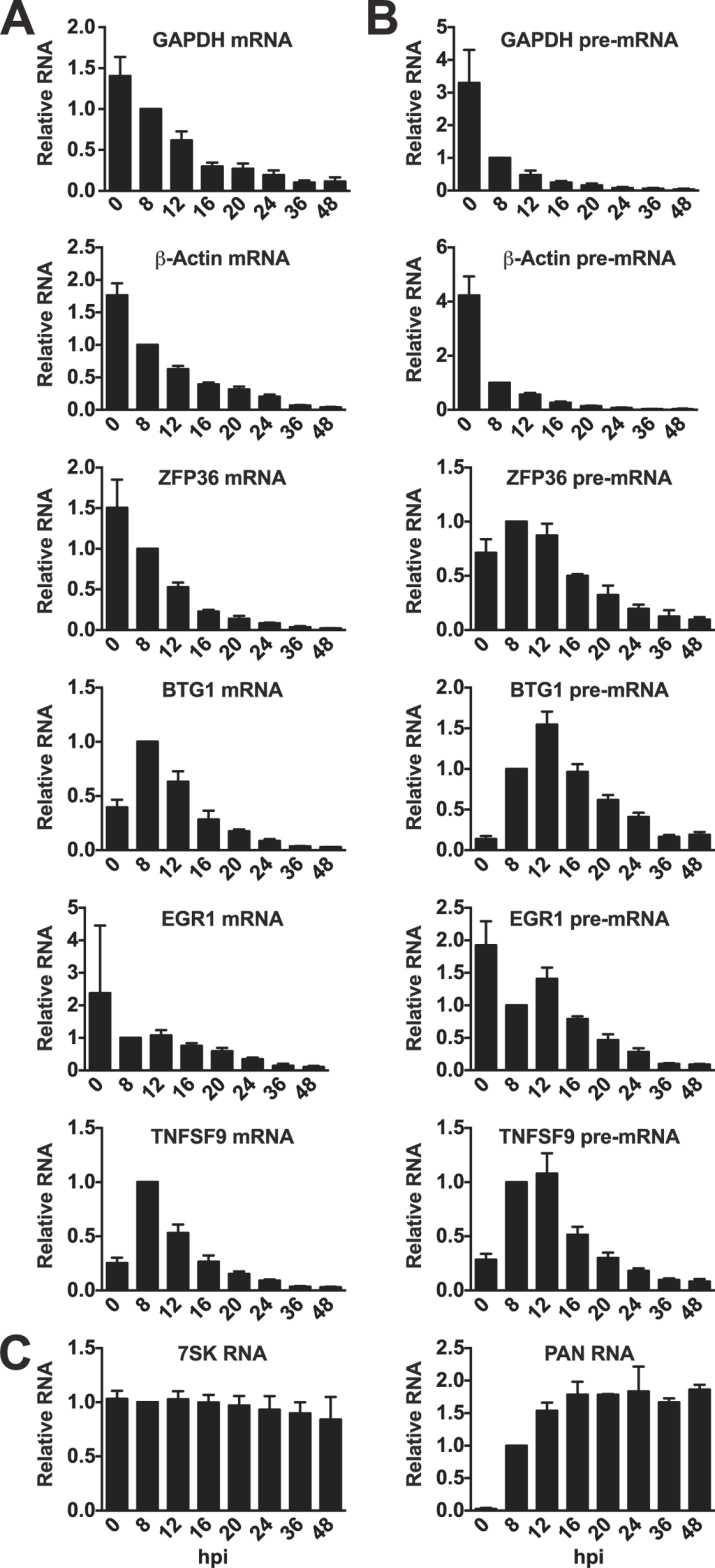
Candidate ORF57-bound pre-RNAs display distinct steady-state level kinetics during lytic reactivation. RNA was harvested at the indicated time points subsequent to lytic reactivation of TREx BCBL1-Rta cells. (A) mRNA, (B) pre-mRNA, or (C) control RNA levels were monitored by RT-qPCR; positions of primers are shown in Fig. 5. Values were first normalized to 7SK RNA and the corresponding value for the 8 hpi time point was set to 1.0. 7SK RNA panel was only normalized to values from 8 hpi, but each experiment used equal amounts of total RNA. Mean values and standard deviation are shown (n = 3).

We next wanted to see if the candidate ORF57-bound host RNAs behaved differently when compared to unbound RNAs during lytic induction. To do so, we monitored transcript steady-state levels at various time points following lytic induction. Upon examination of the mRNA levels of GAPDH and β-actin mRNAs, we saw a marked decrease in transcript levels over time after induction, consistent with these transcripts being subject to RNA decay by KSHV host shutoff (Fig. 6A)[74]. The mRNAs of the ORF57-bound candidates ZFP36, BTG1, EGR1, and TNFSF9 were largely similar to the controls in that each degrades over time after induction. Interestingly, both BTG1 and TNFSF9 were induced ~2.5 and 4-fold, respectively, upon reactivation. This induction was likely due to viral reactivation rather than a nonspecific consequence of butyrate as it was also observed with dox only, albeit at lower levels (S6 Fig.). After this brief induction, the steady-state levels of these mRNAs also decrease. For comparison, we examined two stable nuclear RNAs, the host nuclear noncoding RNA 7SK was unaffected by lytic reactivation and the stable KSHV PAN RNA was robustly induced early in infection but was not lost over time (Fig. 6C).

Enriched clusters were identified in the introns of these RNAs (Fig. 5), so we monitored the pre-mRNA abundance of candidate ORF57-regulated transcripts. Steady-state pre-mRNA levels of transcripts bound by ORF57 followed a different pattern than GAPDH or β-Actin control pre-mRNAs (Fig. 6B). Control pre-mRNAs rapidly disappear: at 8 hours post-induction (hpi) GAPDH and β-actin pre-mRNA levels were reduced by ~4-fold. This rapid disappearance is likely due to splicing of the pre-mRNA, but could also be a result of pre-mRNA decay or even transcriptional shutdown. In contrast, the steady-state levels of pre-mRNAs bound by ORF57 peak at ~12 hpi. In addition, these pre-mRNA peaks occur after the corresponding mRNA begins to decrease (Fig. 6B compared to Fig. 6A). These data demonstrate that the pre-mRNAs bound by ORF57 are subject to different processing and/or decay kinetics during lytic reactivation than unbound controls.

The presence of cellular transcripts made prior to lytic induction, and therefore prior to the presence of ORF57, potentially confounds the interpretation of steady-state analyses. To be sure we are comparing RNA products made prior to ORF57 expression with those generated after ORF57 expression, we employed a metabolic labeling strategy [75,76]. We performed a 2-hr transcription pulse with 4-thiouridine (4SU), a nucleoside analogue readily incorporated into nascent RNAs by all three human RNA polymerases. The presence of 4SU allows us to biotinylate those transcripts generated during the two-hour pulse and these RNAs can then be selected by streptavidin bead purification. Thus, in this assay we specifically monitor the levels of newly made transcripts synthesized during a defined 2-hr period prior to or after KSHV reactivation.

**Fig 7:**
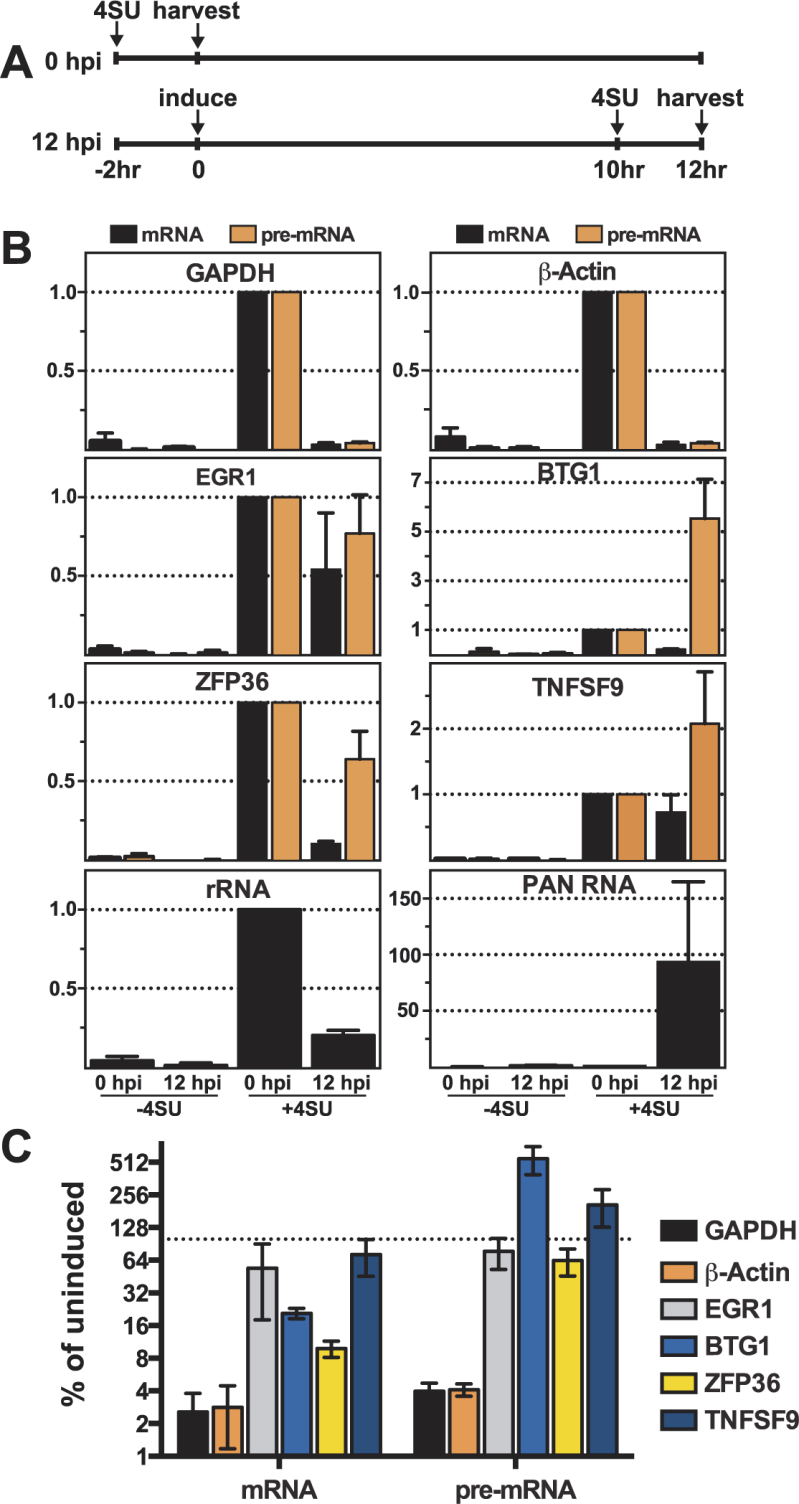
Newly made candidate ORF57-bound pre-RNAs have distinct kinetics during lytic reactivation. (A) Schematic of the time course used for 4SU metabolic labeling experiments. Cells were incubated with 4SU for two hours beginning at -2 and 10 hpi. Cells were collected and RNAs were extracted at 0 and 12 hpi, respectively. (B) Newly made RNA levels were monitored by RT-qPCR with the primers described in Fig. 5. The RT-qPCR values are listed relative to the 0 hpi samples which were set to 1.0. Note that the y-axis scales differ between the panels. The-4SU samples were collected at the same time and processed alongside the other samples, but the cells were not treated with 4SU. Mean values are shown and error bars are standard deviation (n = 3). (C) The data from (B) were plotted for direct comparison between controls (GAPDH and β-actin) and ORF57-bound candidates (EGR1, BTG1, ZFP36, and TNFSF9). The y-axis is on a log scale and the values are presented as the percent relative to the uninduced samples. The dotted line represents 100%.

We compared newly made RNAs collected from uninduced cells (0 hpi) with those from cells at 12 hpi. For the 0 hpi samples, we added 4SU and harvested RNA from uninduced cells 2 hrs later; for 12 hpi samples we added 4SU at 10 hpi and we collected RNA at 12 hpi (Fig. 7A). For GAPDH and β-actin, the levels of newly made mRNA and pre-mRNA decreased after induction of virus (Fig. 7B, top panels). The effect of induction on newly made GAPDH and β-actin RNAs was pronounced: the amount of (pre-)mRNA generated during the two-hour window between 10–12 hpi was only ~2.5–4% of that made prior to lytic reactivation (Fig. 7C). In contrast, none of the pre-mRNAs or mRNAs for the four ORF57 target candidates decreased in such a dramatic fashion. In fact, the pre-mRNAs for BTG1 and TNFSF9 both increased compared to the pre-induction levels (Fig. 7B and 7C), consistent with the apparent induction observed in the steady-state levels (Fig. 6). Newly made EGR1 and ZFP36 pre-mRNA levels were 77% and 64% of the pre-induction levels contrasting with the GAPDH and β-actin controls (Fig. 7B and 7C). Interestingly, the mRNA levels of the candidates were all lower than pre-induction levels. However, they were all significantly higher than the GAPDH and β-actin controls (Fig. 7C). Possible models describing the relationship between ORF57 function, pre-mRNA and mRNA production is explored in further detail below. As an additional control, we showed that newly made PAN RNA is detected only subsequent to induction, as expected (Fig. 7B). Perhaps surprisingly, the levels of newly made ribosomal RNAs also decreased upon lytic induction, but whether this is a virus-induced reduction or a host response to virus has not been determined (Fig. 7B). As an important control, the recovery of RNAs from cells that were not treated with 4SU (Fig. 7B, -4SU) was negligible, thereby demonstrating that our assay is specific for 4SU-containing RNAs. Alongside the steady-state and binding analyses, these data suggest that the metabolism of candidate RNAs ZFP36, BTG1, TNFSF9, and EGR1 is affected by ORF57 and further suggest that ORF57 primarily functions at the pre-mRNA level for these targets. In addition, these observations validate HITS-CLIP as an approach to uncover potential novel functions and targets of ORF57.

### ORF57 is sufficient to up-regulate BTG1, ZFP36 and EGR1 pre-mRNA levels

**Fig 8:**
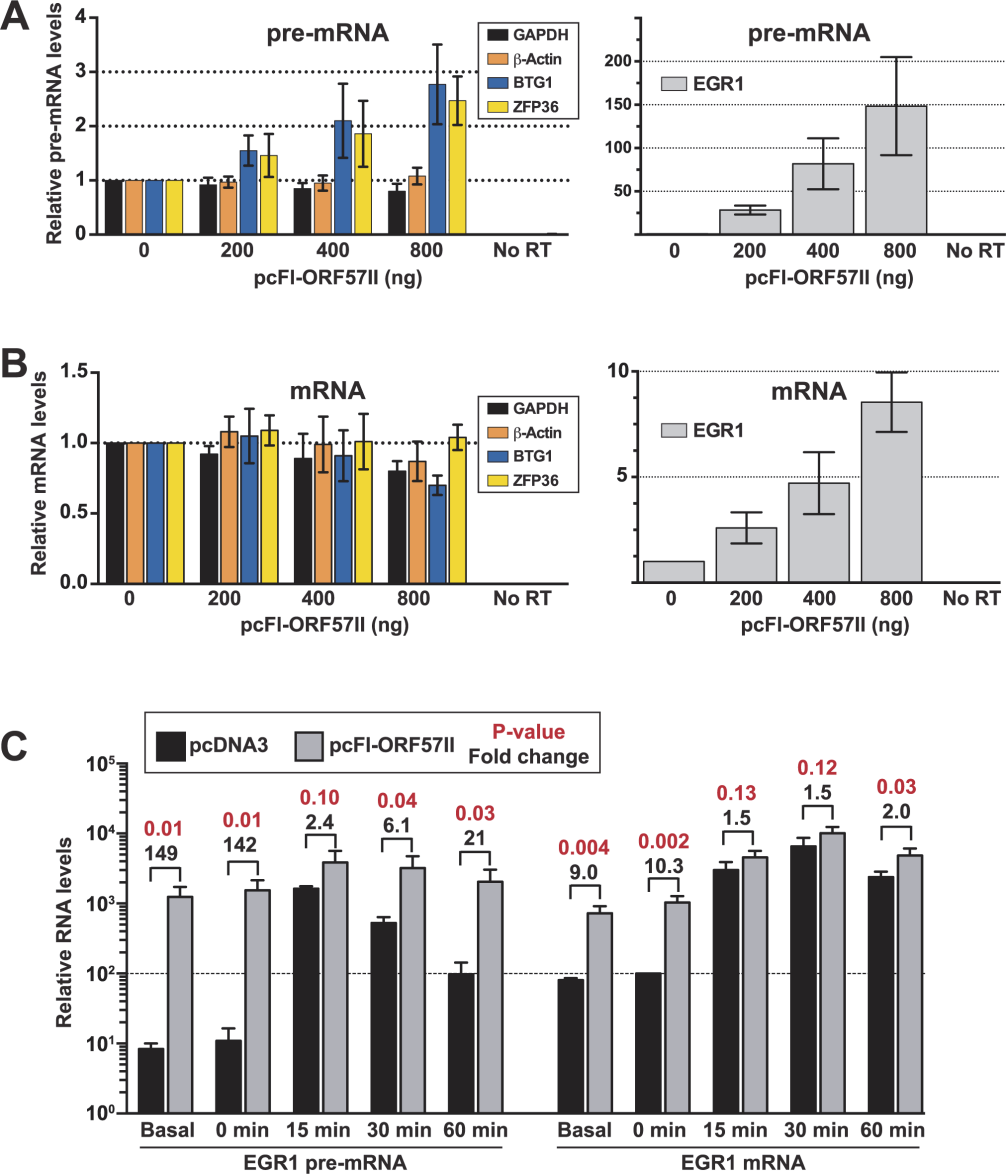
ORF57 is sufficient to up-regulate candidate pre-RNAs in HEK293 cells. (A) An ORF57 expression construct (pc-Fl-ORF57II) or vector control (pcDNA) was transfected into HEK293 cells in the indicated amounts. After 48 hrs, RNA was harvested and RT-qPCR was performed to examine the pre-mRNA levels of GAPDH, β-actin, BTG1, ZFP36 or EGR1. Because of the difference in scale, EGR1 was plotted separately (right panel). Values are shown relative to the vector alone control. (B) Same as (A) except mRNA levels were examined. (C) EGR1 pre-mRNA (left) and mRNA (right) levels were examined after serum starvation and induction as described in the text. The values are the mean of three experiments with standard deviation. The fold differences between pcFl-ORF57II and pcDNA transfected cells are listed above the brackets; p-values for these differences are shown in red (unpaired two-tailed Student’s t-test). Both pre-mRNA and mRNA values are relative to the uninduced (t = 0), vector alone EGR1 mRNA sample, which was set to 100 (dotted line). Therefore, the relative amounts of EGR1 pre-mRNA can be compared to those of EGR1 mRNA in this graph.

Because steady-state levels of the selected candidates were monitored in the context of viral infection, it is possible that changes in their RNA levels were due to the action of viral proteins other than ORF57. Clearly, the contributions of host shut-off to the steady-state and newly made RNA levels (Figs. 6 and 7) cannot be overlooked. To test whether ORF57 is responsible for changes in pre-mRNA metabolism, we monitored the steady-state levels of the BTG1, ZFP36, and EGR1 pre-mRNAs in HEK293 cells 48 hr after transfection with varying amounts of a Flag-tagged ORF57 expression construct (pcFl-ORF57II, Fig. 8A). Unfortunately, TNFSF9 was undetectable in our HEK293 cells, so it was excluded from the analysis. We observed a dose-dependent increase in BTG1 and ZFP36 pre-mRNA levels in the presence of ORF57, but no such increase was observed for the β-actin or GAPDH control pre-mRNAs (Fig. 8A, left). The effects of ORF57 were considerably more dramatic on EGR1 pre-mRNA. At the highest levels tested, we observed an ~150-fold increase in EGR1 pre-mRNA over the vector alone control (Fig. 8B, right). These data show that ORF57 is sufficient to increase the steady-state pre-mRNA levels of BTG1, EGR1, and ZFP36.

So far, we have shown that ORF57 binds (Figs. 4 and 5) and enhances the pre-mRNA levels (Figs. 6–8) of the candidate host genes examined here. We suggest two primary models by which ORF57 could increase pre-mRNA levels that make different predictions regarding the effects of ORF57 on mRNA levels. The first model proposes that ORF57 binds to the pre-mRNA and inhibits splicing, thereby resulting in increased pre-mRNA levels. Supporting this model, the ORF57 homolog ICP27 inhibits pre-mRNA splicing [77–79]. Furthermore, it is easy to imagine that ORF57 binding near exon-intron boundaries (Fig. 4F and 5) would sterically inhibit splicing. In the second model, ORF57 binds pre-mRNAs and inhibits their decay, consistent with its previously ascribed role as a nuclear stability factor [23,24,26,27,31,33]. These models make distinct predictions regarding the effects of ORF57 on mRNA production. Inhibition of splicing would result in a decrease in mRNA levels because the production of mature mRNAs is diminished. In contrast, the second model predicts that mRNA levels will be unaffected or increase upon stabilization of the pre-mRNAs by ORF57. For example, if pre-mRNAs are subject to competition in the nucleus between decay and splicing, stabilization will favor the splicing machinery and increase mRNA production. On the other hand, some pre-mRNAs may simply be “dead-end” products that are no longer substrates for splicing (see Discussion). In this case, ORF57-mediated pre-mRNA stabilization would have little effect on mRNA production.

We therefore examined the effects of ORF57 on BTG1, ZFP36 and EGR1 mRNA levels in transfected HEK293 cells (Fig. 8B). While ZFP36 mRNA levels were unaffected by ORF57 expression, we observed an ~30% decrease in BTG1 mRNA. However, it is important to note that the controls GAPDH and β-actin levels dropped by ~20% and 13%, respectively, so the BTG1 mRNA decreases seem unlikely to be specific. In contrast, EGR1 mRNA levels increased in a dose-dependent fashion upon ORF57 expression, but not to the same extent as the pre-mRNA (8.5-fold compared with 148-fold). A reasonable explanation for these data is that a significant fraction of EGR1 pre-mRNA is inefficiently spliced and degraded under normal conditions. However, ORF57-mediated stabilization of the EGR1 pre-mRNA permits splicing of some of the stabilized pre-mRNAs resulting in increased mRNA production.

This model proposes that under normal cell culture conditions EGR1 pre-mRNAs are inefficiently spliced and degraded. Importantly, cells tightly regulate EGR1 expression by rapidly inducing EGR1 upon re-addition of serum to serum-starved cells [80]. We reasoned that cells would increase EGR1 pre-mRNA splicing efficiency to maximize EGR1 mRNA production under inducing conditions. If this assumption is correct, then ORF57 is predicted to have less of an effect on induced EGR1 pre-mRNA and mRNA levels after serum induction because the cells shift the competitive balance between pre-mRNA splicing and decay to favor pre-mRNA splicing. To test this idea, we transfected HEK293 cells with pcFL-ORF57II or an empty vector control (pcDNA). Approximately 36 hours after transfection, we replaced the media with serum free media to serum starve the cells overnight. The next morning, we added serum back to the media and harvested RNA from cells 0, 15, 30, and 60 minutes following serum induction. We then compared the levels of EGR1 mRNA and pre-mRNA (Fig. 8C). The serum-starved EGR1 levels (0 min) were nearly identical to the non-serum starved samples (basal). As expected, ORF57 had a robust, statistically significant effects of ~140-fold and ~10-fold for the pre-mRNA and RNA, respectively. In contrast, 15 minutes following induction the presence of ORF57 increases the pre-mRNA and mRNA levels by 2.4 and 1.5-fold, respectively and these changes are not statistically significant (p-value>0.05). In the control samples, the pre-mRNA levels begin to diminish after the 15-minute time point, but the ORF57 expressing cells maintain a high level of pre-mRNA. The mRNA levels remain comparable between the samples for up to 60 minutes, likely due to persistence of the induced mRNA in the cytoplasm. These observations further support the model that ORF57 binds and stabilizes a subset of host pre-mRNAs. In addition, these data validate the results of our HITS-CLIP analysis and strongly suggest that ORF57 modulates the processing of host (pre-)mRNAs in addition to its roles in viral gene regulation. Furthermore, they suggest that the consequences of ORF57-mediated stabilization depend on the nuclear processing and decay rates of the specific bound transcript.

## Discussion

### HITS-CLIP identification of ORF57-bound RNA fragments

In this study, we performed HITS-CLIP of the KSHV ORF57 protein in a lytically reactivated PEL cell line to globally identify ORF57-bound RNA fragments. We recovered enriched clusters that map to both human and viral genomes (Figs. 2–5). For the viral RNAs, our results were validated by the identification of the known ORF57 binding site at the 5´ end of PAN RNA [26–28]. In addition, our previous ChIP study showed that ORF57 interacts with the KSHV genome near oriLyt-L [46], but ChIP assays do not distinguish between direct interactions with DNA, indirect interactions mediated through other DNA-bound proteins, or indirect interactions bridged via nascent RNA. Our discovery here of enriched clusters over the RNAs overlapping with oriLyt-L (Figs. 2 and 3) strongly supports the hypothesis that the ChIP signals were due to interactions with RNA. Interestingly, the ORF57 ChIP signal across the oriLyt-L was not lost upon RNase treatment [46]. In addition, we observed putative ORF57 multimers bound to the same RNA that were resistant to high levels of MNase (Fig. 1). Further supporting multimerization, ~50% of the human genes had more than one enriched cluster per transcript S5 Fig.). This represents a minimum estimation because a single enriched cluster could possibly represent more than one binding site, whereas two enriched clusters are unlikely to arise from one ORF57 binding event. Together, these data suggest that ORF57 binds to nascent transcripts, multimerizes, and forms a complex containing relatively inaccessible RNA. We also observed enriched clusters at the oriLyt-R region (S1 Table) supporting a general function for ORF57 at these sites. How and whether this binding relates to KSHV DNA replication is unknown, but oriLyt-associated transcription is essential for DNA replication [81] and this function is conserved in EBV [82].

The identification of known ORF57 targets and the identification of novel host RNAs affected by ORF57 validate our approach. Admittedly, our analysis is not without its limitations. HITS-CLIP data presents bioinformatic challenges and various methods have been applied to filter bona fide bound fragments from background (discussed in reference [83]). The condensed viral genome presents potential additional challenges, which could obscure the identification of viral enriched clusters. For example, if ORF57 binds to a majority of viral transcripts, the density of clusters in the pellets would be higher overall and equivalent among transcripts, so specific binding events may not be identified as enriched. In fact, we needed to decrease the stringency to identify at least one known ORF57 target (ORF59, Fig. 3). We chose to define ORF57 binding sites by examination of the pellet tag counts relative to the input samples. The advantage of this approach is that it provides a robust control for expression levels so that highly expressed genes are not overrepresented in the data set. As described in more detail below, this approach may bias against the identification of stable efficiently processed RNAs if ORF57 is removed from transcripts at later stages of RNA metabolism. We do emphasize that these caveats would lead to a potential underrepresentation of the number and scope of RNAs bound by ORF57 and highlight that the definition of enriched clusters employed herein is conservative. Importantly, the raw data are publically available (NIH GEO database, GSE64413), so independent researchers can apply bioinformatic pipelines using our data. In summary, the validation of our novel targets and identification of previously known targets support the conclusion that we have generated a robust data set, but we expect that further data mining will yield additional information regarding ORF57 targets.

### Novel human ORF57 pre-mRNA targets

**Fig 9:**
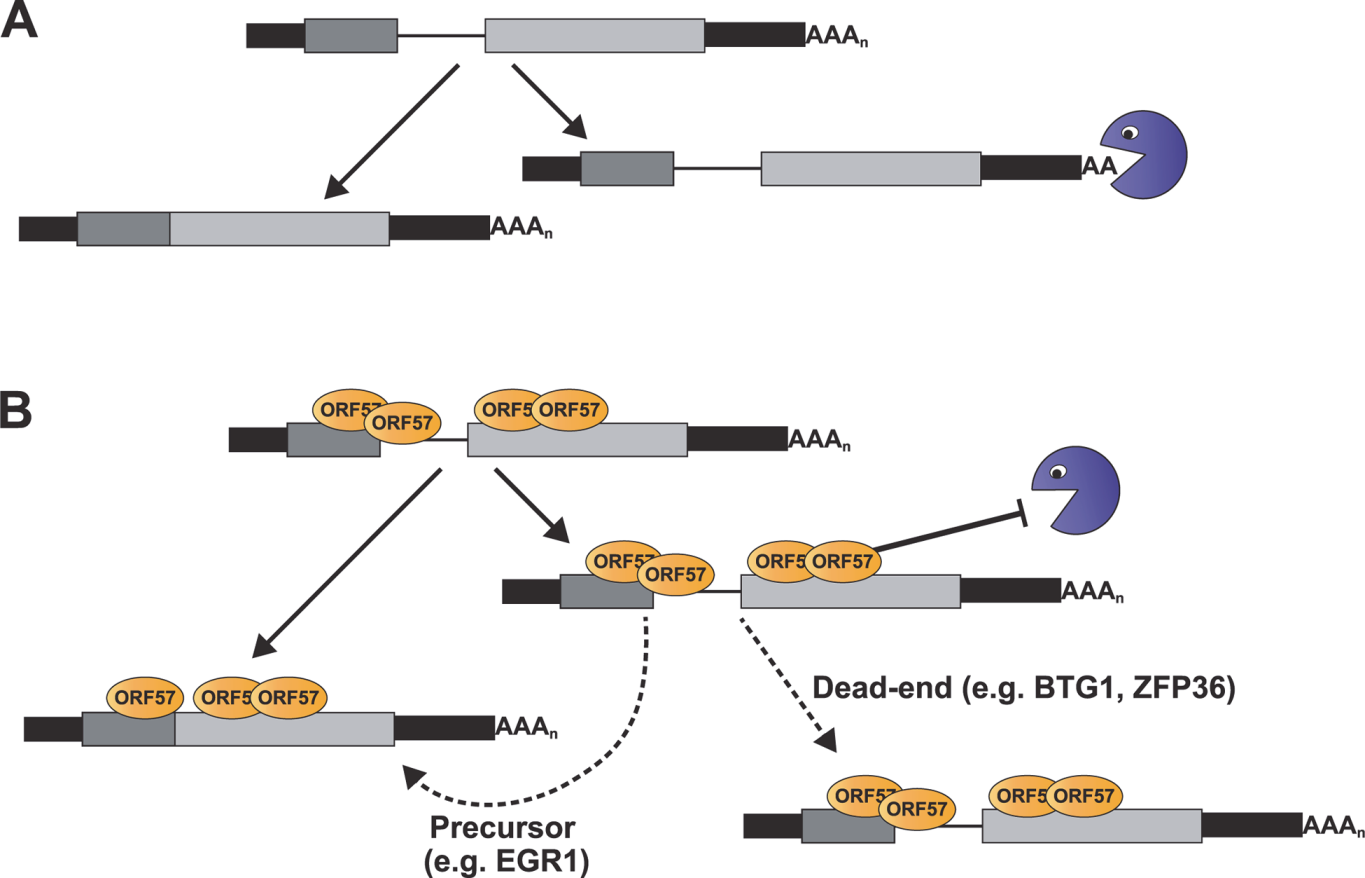
Model for ORF57-mediated regulation of host pre-mRNAs. (A) Models of pre-mRNA and mRNA balance for host ORF57 targets in (A) absence or (B) presence of ORF57. While ORF57 is the only protein depicted, it is likely to be in complex with other host proteins, (e.g. TREX, SR proteins, the cap-binding complex). The pacman represents an unidentified host RNA decay pathway. Briefly, the host pre-mRNAs are subject to competing RNA decay and splicing pathways (A). When bound by ORF57 (B), the pre-mRNAs are stable which results either in the accumulation of pre-mRNAs (“Dead-end”) or their stabilization allows these pre-mRNAs to be further spliced to produce functional mRNAs (“Precursor”). See Discussion for the details of the models.

We identified a subset of enriched clusters that showed enhanced ORF57 binding at the 5´ ends of host transcripts, near the first exon-intron boundary (Fig. 4). Examination of the levels of four of these RNAs during lytic reactivation demonstrated altered kinetics of these pre-mRNAs compared to control pre-mRNAs (Figs. 6 and 7). Importantly, ORF57 expression was sufficient to increase the pre-mRNA levels of BTG1, ZFP36, EGR1, as well as EGR1 mRNA, but had little effect on BTG1 or ZFP36 mRNA abundance. We can imagine several models of ORF57 activity on these RNAs that are not mutually exclusive. Our preferred model proposes that some cellular pre-mRNAs are not constitutively spliced, but rather are subject to either splicing or decay in the nucleus (Fig. 9A). ORF57 binds the inefficiently spliced pre-mRNAs and stabilizes them thereby increasing pre-mRNA, and in some cases, mRNA levels (Fig. 9B). This model proposes no new mechanisms for ORF57, but extends its previously described activities in nuclear viral RNA stability to host pre-mRNAs. We further suggest that the distinct effects on mRNA levels (Fig. 8B) derive from the cellular metabolism of the specific pre-mRNAs and are not due to an activity of ORF57 directly. In the case of EGR1, the splicing and decay machineries are in kinetic competition with each other so increases in pre-mRNA stability shift the competitive balance such that more pre-mRNA splicing occurs (Fig. 9B, “Precursor”). In contrast, BTG1 and ZFP36 unspliced pre-mRNAs are fated to be discarded by a pre-mRNA decay pathway and are not subject to further pre-mRNA splicing upon stabilization; thus, their pre-mRNA levels increase with no concomitant increase in mRNA (Fig. 9B, “Dead-end”). As a result, the biological relevance of ORF57-mediated stabilization of BTG1 and ZFP36 pre-mRNA is not immediately obvious because ORF57 appears stabilize dead-end products. Perhaps in other cell types stabilized BTG1 and ZFP36 pre-mRNAs are mRNA precursors similar to EGR1. In fact, newly made BTG1 and ZPF36 mRNA levels remain higher than GAPDH or β-actin at 12 hpi (Fig. 7C), consistent with the idea that the stabilized pre-mRNAs are converted to mRNAs in lytically reactivated TREx BCBL1-Rta cells. Overall, should this model prove true, these studies lay the foundation for a new role for ORF57-mediated RNA stabilization, and they provide additional insight into the relationship between processing and nuclear decay of cellular pre-mRNAs.

If ORF57 protects both viral and cellular RNAs from host nuclear RNA decay factors, then it is of interest to identify the cellular RNA decay pathway involved. We have recently identified a nuclear RNA decay pathway that is dependent on the nuclear poly(A)-binding protein, PABPN1, and the poly(A) polymerases PAPα and PAPγ [27,28,41,84]. Several lines of evidence suggest that ORF57 inhibits this pathway. First, PABPN1-dependent decay is responsible for the degradation of an unstable allele of PAN RNA and ORF57 is sufficient to stabilize this same allele of PAN RNA [27,28,41,84]. Second, substrates for PABPN1-dependent decay must be polyadenylated and display a longer nuclear dwell-time, as may be expected for intronless viral RNAs and inefficiently spliced pre-mRNAs [41,85]. Third, our unpublished studies suggest that EGR1 levels increase when this pathway is inhibited (S. Bresson and NKC). Therefore, we speculate that ORF57 protects inefficiently processed pre-mRNAs from PABPN1-mediated nuclear RNA decay and our ongoing studies seek to directly test this hypothesis.

In an alternative model, increases in pre-mRNA levels result from inhibition of splicing by ORF57. Consistent with this idea, the herpes simplex virus ORF57 homolog ICP27 inhibits splicing [77–79]. In addition, it is easy to imagine that the presence of ORF57 at the 5´-most splice site (Fig. 4) would occlude the splicing machinery. In fact, we observed a slight decrease in BTG1 mRNA levels in ORF57 expressing cells, but GAPDH and β-actin were also reduced so the specificity of this effect is questionable (Fig. 8B). We think this model less likely than the stability model for several reasons. First, it is difficult to reconcile the splicing inhibition model with the observed increases in EGR1 mRNA levels. Second, a previous report showed that, unlike ICP27, ORF57 enhances the splicing of some transcripts [42,86] and the Epstein-Barr virus (EBV) homolog of ORF57, SM, also modulates pre-mRNA splicing [87,88]. Third, we consider the stability model to be the simplest mechanism as it involves a previously documented function for ORF57.

While we favor the stability model, we are cautious in our interpretations of the mechanisms driving the changes in steady-state levels of pre-mRNA and mRNA. The pre-mRNA-to-mRNA ratio is often taken as a measurement of splicing efficiency. However, when one considers nuclear pre-mRNA decay as a contributing factor, interpretation of this ratio becomes muddled. For example, in our preferred model (Fig. 9), the increases in pre-mRNA-to-mRNA ratios for dead-end transcripts are attributed to stabilization rather than inhibition of splicing. For “precursor” RNAs the situation is even more complex. If the mRNA is not particularly stable, one will detect increases in the pre-mRNA-to-mRNA ratio due to the longer half-life of the pre-mRNA. In contrast, if the resulting mRNA is very stable in the cytoplasm, then the steady-state pre-mRNA-to-mRNA ratio will decrease because the mRNA accumulates. Of course, this complexity does not mean that changes in pre-mRNA-to-mRNA ratios observed upon ORF57 expression are not due to alterations in splicing [42,86], but we emphasize that distinction between modulation of splicing versus nuclear RNA decay represents an empirical challenge that can not be discerned solely from pre-mRNA-to-mRNA ratios. Thus, while splicing inhibition is not our preferred explanation for ORF57-mediated up-regulation of host pre-mRNAs, we cannot exclude this hypothesis without further experimentation.

In principle, our data are also consistent with a role for ORF57 in inducing transcription of these targets, particularly in the case of EGR1. The EGR1 response to ORF57 in HEK293 cells (Fig. 8C) does not perfectly mimic EGR1 induction by serum, but it is similar at the pre-mRNA level. Because EGR1 induction is likely at the level of RNA synthesis [64,65,89,90], it remains possible that ORF57 is initiating this transcriptional response. If ORF57 induces EGR1, it is not fully induced as there is still an ~10-fold induction of EGR1 mRNA in the presence of ORF57 (compare mRNA levels at 0 with 30 min, Fig. 8C). While this model seems unlikely given ORF57’s known roles in posttranscriptional gene regulation and its binding to EGR1 (Fig. 5), ORF57 has been implicated in transcription of some viral genes [34,46,47]. Moreover, the documented cross-talk between RNA-binding proteins, the spliceosome and the transcription machineries suggest that this model must not be overlooked [91–97]. Further dissection of ORF57 mechanisms is necessary to distinguish among these models.

### How is ORF57 recruited to its RNA partners?

ORF57 is sufficient to increase the abundance of intronless RNAs including viral mRNAs, PAN RNA, and intronless reporters. In the case of PAN RNA and vIL6, this up-regulation is enhanced by the presence of the specific cis-acting sequences that appear to serve as specific ORF57 binding sites [26,28,43]. However, ORF57 binding is not restricted to the ORE or ORE-like elements. In fact, given its ability to up-regulate a wide variety of nonviral reporters of no biological relevance for ORF57, ORF57 must be recruited to RNAs in a general fashion. When we analyzed the enriched cluster sequences using MEME, a *de novo* motif discovery algorithm [98], we were unable to retrieve significant conserved elements. Therefore, it remains possible that its specific binding to PAN RNA and vIL6 are exceptional and that ORF57 is recruited to RNAs in a largely sequence non-specific fashion. Interestingly, a similar balance between general and specific RNA-binding has been proposed for the EBV SM protein [99] supporting a conserved mode of RNA binding between the gammaherpesvirus posttranscriptional regulators of gene expression.

How could ORF57 achieve apparent specificity in binding and function if it has little or no sequence specificity? ORF57 has been reported to interact with a number of host RNA-binding proteins, so it is possible that these proteins dictate the apparent specificity of binding [16–18,25,37,55] or regulation of local concentrations of ORF57 could contribute to binding preference as well. Alternatively, ORF57 may transiently bind nearly all newly made RNAs. In the case of efficiently processed RNAs (e.g. GAPDH, β-actin), the binding has little consequence because the pre-mRNAs are quickly spliced, exported and translated. If ORF57 is removed from the RNA during splicing, export, or translation, we would not have identified an enriched clusters because the ratio of ORF57-bound immature RNA to the unbound fully processed cytoplasmic mRNA would be relatively low. In contrast, an inefficiently processed cellular (pre-)mRNA would remain engaged with ORF57 in the nucleus and therefore be more likely to be identified as an enriched cluster due to its higher ratio of bound to unbound RNAs in the cell. In addition, transcripts with a longer nuclear dwell time will display an apparent (but indirect) functional specificity due to ORF57-mediated protection of those transcripts from nuclear decay. That is, efficiently exported RNAs are not subject to nuclear decay and are thus unaffected by ORF57 binding in the nucleus. In contrast, those transcripts that have a longer nuclear dwell time would normally be subject to degradation, but ORF57 protects them and increases their abundance.

### Conclusions

Because of its critical role in the viral life cycle, a mechanistic understanding of ORF57 functions is essential to the understanding of KSHV replication and pathogenesis. Our high throughput screening of ORF57-bound RNAs begins to address interactions of ORF57 with viral and host RNAs. This work extends existing data supporting a general role for ORF57 in the stabilization of a wide variety of viral RNAs. In addition, these data suggest that ORF57 nuclear RNA stabilization function is not restricted to viral RNAs, but further modulates the processing and decay of host transcripts during lytic reactivation. Ongoing studies seek to identify the precise molecular mechanisms of ORF57 interactions with the host cell RNA decay machinery that promote the stabilization of viral and host transcripts in the nucleus. In addition, it is of great interest to determine how changes in gene expression induced by ORF57 binding of host RNAs affect viral replication and/or pathogenesis.

## Materials and Methods

### Cell culture, transfection, and RT-qPCR

TREx BCBL1-Rta cells [53] were carried in RPMI-1640 media (Sigma) supplemented with 10% tetracycline-free fetal bovine serum (FBS, Clontech), penicillin-streptomycin (Sigma), 2 mM L-glutamate, and 100 μg/ml hygromycin (Sigma). Lytic reactivation was induced by addition of 1μg/ml doxycycline and 3 mM sodium butyrate. HEK293 cells were grown in DMEM media (Sigma) supplemented with 10% FBS (Sigma), penicillin-streptomycin, and 2 mM L-glutamate. HEK293 cells were transfected using TransIT-293 (Mirus) according to the manufacturer’s protocol. The flag-tagged ORF57 expression vector was previously described (pcFl-ORF57II, [27]). For the ORF57 dose-dependency experiments (Fig. 8A and 8B), HEK293 cells were transfected in a 12-well plate with a combination of pcDNA3 and pcFl-ORF57II totaling 800 ng. Twenty-four hours after transfection the cells were ~100% confluent and transferred to 6-well plates. The next day (48 hrs post-transfection), cells were ~70–80% confluent and RNA was harvested in TRI-Reagent (Molecular Research Center). For serum starvation and induction experiments (Fig. 8C), we transfected 6 μg of pcDNA3 or pcFl-ORF57II in a 60-mm plate. The following morning, we split these cells evenly among five wells of a 6-well plate. Approximately 10 hrs later, cells were ~70–80% confluent and we replaced the media with serum free media. The next morning (48 hrs post-transfection), we added serum to 20% and harvested as described. We stress that cell confluency and freshness of the media were crucial for reproducibility in the HEK293 transfection experiments. RT-qPCR assays were performed with standard techniques using iTAQ Universal SYBR Green Supermix (Bio-Rad) with a final primer concentration of 100 nM. The conditions were 40 cycles of 95°C for 3 sec and 60°C for 30 sec with a 7500 Fast real-time PCR system (Applied Biosystems). Random hexamers were used for first-strand synthesis and gene-specific primers are given in S3 Table.

### HITS-CLIP lysate preparation and immunoprecipitation

HITS-CLIP was performed essentially as previously described [51,52], but changes were made to maximize the solubility and recovery of ORF57. For each immunoprecipitation a total of 2x10^7^ cells were reactivated at 5x10^5^ cells/ml and then collected at 20 hpi. Cells were washed in phosphate buffered saline (PBS, Sigma), and resuspended in 3 mL of ice-cold PBS. Crosslinking was performed on ice in a Spectrolinker (Spectronics Corporation) at 125mJ/cm^2^ ~2 cm from the 254nm UV bulb. Five separate immunoprecipitations were performed for each biological replicate for a total of 10^8^ cells per pellet sample. Cells were pelleted, frozen on dry ice and stored at -80°C.

Lysates were generated by previously described procedures [49] with a few changes. The composition of the SDS lysis buffer was 0.5% SDS, 50mM Tris pH 6.8, 1mM EDTA, 0.125 mg/ml Heparin, 1mM DTT, 1mM PMSF and 1X protease inhibitor (Calbiochem), while RIPA correction buffer was 1.25% NP40, 0.625% sodium deoxycholate, 62.5 mM Tris pH 8.0, 2.25 mM EDTA, 187.5 mM NaCl, 0.125 mg/ml Heparin, 1mM DTT, 1mM PMSF, with 1X protease inhibitors. Initial steps of extract preparation were as described [49] up to and including initial shearing of DNA with a QIAShredder (Qiagen). Next, CaCl_2_ was added to 5 mM with 30 U of RQ1 DNase (Promega) and incubated for 15 min at 25°C. For RNA digestion, MNase (New England Biolabs) was diluted to 10 gel units/μL in RIPA buffer and 5 μL of this freshly diluted 1:200 stock was added to the extract. No dilution was performed for the “high MNase” samples (Fig. 1). RNA digestion proceeded at 25°C for precisely 10 min, after which 47 μL of 300mM EGTA was added to stop the reaction. After clarification of the lysate by three successive centrifugation steps at 21K x g for 10 min, the lysate was pre-cleared and the ORF57-RNA complexes were immunoprecipitated with protein A Dynabeads (Invitrogen). The ORF57 antibodies [28] were affinity-purified from rabbit serum using the AminoLink Plus Immobilization kit (Pierce) as per the manufacturer’s instructions. We used 48 μg of purified antibody per immunoprecipitation. The immunoprecipitated complexes were washed twice with RIPA Buffer, twice with high salt buffer (5X PBS, 0.1% SDS, 0.5% NP40), and twice with 1X PNK Buffer.

### HITS-CLIP ORF57-RNA isolation, library preparation and sequencing

Gel purification of complexes was performed essentially as described in [51,52]. Briefly, immunoprecipitated cross-linked RNAs were 5´-end-labeled with PNK and γ^32^P-ATP. The protein-RNA complexes were resolved in a 4–12% Bis-Tris NuPAGE Novex gel and transferred to a nitrocellulose membrane. Covalently bound, ORF57-RNA complexes were cut from the membrane, treated with proteinase K and the recovered RNAs were size selected in a denaturing urea gel. For each biological replicate, the RNAs from five immunoprecipitation reactions were combined prior to library preparation. For input samples, total RNA was recovered with TRI-Reagent at 20 hpi and rRNAs were depleted using the Ribo-Zero Magnetic Kit (Epicentre). Input RNAs were treated with MNase, end-labeled, and size selected prior to library preparation to mirror the pellet samples. Libraries were made according to Illumina’s TruSeq Stranded mRNA Sample Preparation Guide, but the fragmentation step was omitted. Sequencing was performed on an Illumina HiSeq 2500 sequencer at the Eugene McDermott Center for Human Genetics. A detailed step-by-step lab protocol for HITS-CLIP with ORF57 can be obtained by contacting the corresponding author (NKC). Raw sequencing reads for all biological samples in this study are available, along with processed data files, under GEO accession number GSE64413.

### Sequence data trimming and mapping

Adaptors marked as N at the 3’ends of the paired-end sequencing data were first trimmed. Then Gsnap was used to align the sequencing data with parameters “-A sam—maxsearch 1-N 1-t 4-n 1” [100,101]. The reads were aligned to the concatenated hg19 and U75698.1 genomes. Reads that mapped across splice-junctions were discarded. Mapping statistics are provided in S4 Table. Unaligned reads by Gsnap were subsequently mapped to the hg19 transcriptome by Novoalign (Novocraft).

### CLIP tag clustering

Paired-end read pairs were merged into single-end format if they overlap by at least 1 bp. Merged reads that have the same chromosome, strand, left-most mapping coordinate and right-most mapping coordinate were defined as PCR amplification duplicates and collapsed to unique tags, keeping only the tag with the highest sequencing quality. Then tags from all three pellet and three input experiments were simultaneously overlapped to identify CLIP clusters with at least 10 tags from any one or more conditions. CLIP clusters were continuous regions with non-zero binding tag count on each base within the clusters. Then the CLIP clusters were binned by 20bp and the tag counts and mutation counts on each base were summed for each bin within each experimental condition separately.

### Enriched cluster detection

Deletions and T→C mutations were chosen as characteristic RT-induced mutations of ORF57 binding according to comparative analysis of all the 12 types of substitutions, deletions and insertions (see below). The summed total tag count and summed characteristic mutation count within each bin were multiplied by 0.8 and multiplied by 0.2, respectively, and further summed to yield a value we termed the overall binding intensity for that bin. As a result, each bin will have 6 overall binding intensity numbers corresponding to the three pellet and three input samples.

We used DESeq to analyze the binding intensity data of the three pellet vs. three input samples [102]. For DESeq analysis, we used the median intensity value of each condition for normalization and employed a negative binomial test for identifying differentially bound regions between the pellets and input samples. The analysis was conducted separately for human binding sites and virus binding sties and a p-value was assigned for each bin. Bins that were more enriched (p-value<0.001) in the pellets compared to the inputs were extracted and neighboring enriched bins were concatenated into continuous regions indicating the highly reliable ORF57 binding sites. The binding sites were screened to retain only those sites that span at least 3 bins and have on average at least four tags in the pellet samples. In addition, the ORF57 binding sites that overlap repeating sequences including rRNAs, tRNAs, low complexity regions, LINEs, SINEs and simple repeats were discarded [103]. The remaining binding sites are referred to as enriched clusters.

### Definition of ORF57 induced characteristic mutations

In order to identify reverse transcription mutations that are characteristic of ORF57 crosslinking sites [57], we performed several analyses (S7 Fig.). First, the mutations on all the sequencing tags for all biological replicates were summed for both the pellet and input samples, and a ratio of pellet/input mutations was calculated for each mutation type. The higher this ratio, the more mutation counts of the given type there are in the pellet samples compared to the input samples (S7A Fig.). In this case, the deletion mutations dominated, followed by insertions and T→C mutations. However, it is important to note that this analysis does not consider the absolute number of mutations, which differed considerably among the mutation types, particularly in the input samples (S7B Fig.). More importantly, T→C and A→G mutations, rather than deletion mutations, are the most abundant mutation in the HITS-CLIP pellets (S7B Fig.). The sequencing depths of HITS-CLIP and input samples were further considered to yield a per-tag rate for each mutation type (S7C Fig.). In this case, deletion mutations showed the largest increase when we compared pellet to input, but T→C and A→G mutations have the larger absolute mutation rates in HITS-CLIP samples. Finally, ORF57 crosslinking should lead to both sequencing tag and characteristic mutation enrichment around binding sites, so the total tag count in the pellets should display a positive correlation with the characteristic mutation count. For our analysis, the sequencing tags were clustered and binned into 20-bp units. Total tag counts as well as mutant tag counts were profiled on the bin-level for all clusters. The correlation was calculated between the bin-level total tag count and each type of mutant tag count data (S7D Fig.). Deletion, T→C and C→T mutations have the largest increases in Pearson correlation in pellet samples compared with the input controls. Based on all of these analyses, we chose deletion and T→C mutations as characteristic mutations for the ORF57 protein binding sites. Mutant bases with deletions and T→C mutations were extracted from within the enriched clusters. T-tests with p-value cutoff 0.05 are applied to test whether the mutant bases have significantly higher rates of mutant tag vs. total tag ratio in the pellets. The genome coordinates for enriched cluster mutations with p-value<0.05 are given in S5 Table.

### Annotation analysis

Enriched clusters mapping to the human genome were annotated by genomic features including coding sequence (CDS), 3´ UTR, 5´ UTR, intron, 2kb upstream region, 2kb downstream region, miRNA/snoRNA and intergenic region. 2kb upstream region represents 2kb sequences before all genes’ transcription start sites. 2kb downstream region represents 2kb sequences after all genes’ transcription stop sites. Intergenic regions were defined as those parts of the genome that do not belong to any of the other categories listed here. If a cluster or a tag overlaps more than one feature, its count were divided proportionally and added towards each of the corresponding feature categories.

### Metabolic labeling with 4SU

Metabolic labeling and isolation of newly made RNAs were modified from previous protocols [75,76] Twenty micrograms of DNase-treated RNA was biotinylated in a 50 μl reaction containing 10mM TrisHCl (pH 7.5), 1mM EDTA, 0.1% SDS, and 0.2mg/ml EZ-Link Biotin-HPDP (ThermoScientific). The reaction was incubated for 3 hr at room temperature. RNA was extracted twice with chloroform and ethanol precipitated in 1M ammonium acetate with 15 μg of glycoblue (Ambion) as a carrier. Streptavidin selection was carried out with 20 μl of the bead slurry of Dynabeads MyOne Streptavidin T1 (Invitrogen). The beads were pre-washed in MPG1:10-I buffer (100mM NaCl, 1mM EDTA, 10mM Tris pH 7.5 and 0.1% Igepal). After the last wash, the beads were resuspended in 180 μL of MPG1:10-I and pre-blocked with 0.1 μg/μl poly(A) RNA, 0.1 μg/μl salmon sperm DNA, 0.1 μg/μl of Torula yeast RNA (Sigma). After precipitation, the biotinylated RNA was resuspended in 30 μl of water. RNA was heated at 65°C for 5 min and subsequently nutated with 170 μl of the blocked beads for 30 min at room temperature. The binding reaction was then washed with 300 μl of: MPG1:10-I, MPG1:10 (100mM NaCl, 1mM EDTA, and 10mM TrisHCl (pH 7.5)) at 55°C, MPG1:10-I, MPG-I (1M NaCl, 10mM EDTA, 100mM Tris pH 7.5 and 0.1% Igepal), MPG-I, MPG1:10-I, MPG-I no salt (10mM EDTA, 100mM Tris pH 7.5 and 0.1% Igepal) and MPG1:10-I. The bound RNAs were then eluted by incubating for 5 min in 200 μl of MPG1:10-I with 5% β-mercaptoethanol first at room temperature then a second elution was performed in the same buffer at 65°C for 5 min. Eluted fractions were combined, phenol:chloroform:isoamyl alcohol (25:24:1) extracted, chloroform extracted, and then ethanol precipitated with sodium acetate and 15 μg of glycoblue. RT-qPCR was performed to detect specific RNAs.

## Supporting Information

S1 Fig Characterization of RNA fragments recovered from nitrocellulose membrane. (A) The 37-kDa, 50-kDa and larger (combined 100- and 150-kDa) protein-RNA complexes were cut from the nitrocellulose membrane, proteinase K treated and the purified RNAs were run on a denaturing urea gel. RNAs isolated from the condition treated with high MNase concentrations are loaded on the left, while the fragments digested with low MNase concentrations are on the right. Mobility of a DNA ladder (nt) is shown on the right. (B) RNAs digested with a low MNase concentration were extracted from the 37-kDa, 50-kDa and combined 100- and 150-kDa protein-RNA complex bands. These RNAs were used for northern blot analysis and probed for PAN RNA (B) or 5.8S rRNA (C). Positions of bands from a DNA ladder are included on the left of each membrane.
(EPS)

S2 Fig Scatter plot and Pearson’s correlation coefficients for input and pellet samples. Scatter plots are shown on the upper right and Pearson’s correlations are shown on the lower left. For scatter plots, the CLIP tags in the human and KSHV genomes were binned (20 bp/bin) and the raw tag count data was log transformed as log_e_(raw tag count + 1). Pearson’s correlations represent the correlation between raw tag counts of any 2 conditions.
(EPS)

S3 Fig Genomic location of KSHV enriched clusters from the low stringency dataset. The x-axis represents position on the KSHV genome (U75698) and the midpoint of each cluster was used as the x-coordinate. In this dataset, the KSHV enriched clusters ranged from 19–4339 nt; the mean viral enriched cluster length was 209 nt and the median was 99 nt. A statistical cutoff of 0.05 was used to define enriched clusters (dashed lines). For display, the RNA fragments mapping to the KSHV plus strand were assigned -log_10_(p-values) (black) while the minus strand clusters are displayed as log_10_(p-values) (orange).
(EPS)

S4 Fig Characterization of enriched clusters mapping to the human genome in the low stringency dataset. (A) Pie chart of the gene feature annotations of human enriched clusters from the low stringency dataset. If a cluster spanned multiple different annotations, the cluster annotation was split proportionally among the annotations. Upstream and downstream 2 kb refer to clusters mapping within 2 kb immediately flanking the nearest annotated gene. (B) The bar graph compares the number human enriched clusters (ECs) relative to their position on the closest annotated transcript. 0.0 and 1.0 correspond to the transcription start site (TSS) and the poly(A) site, respectively. Importantly, the length in this graph is relative to the annotated gene and is not a measure of the distance in base pairs. The brackets denote the 5´-enriched clusters examined in C-E. (C) Pie graph classifies the low stringency 5´-enriched clusters based on their gene feature annotations. (D) Bar graph comparing the number (y-axis) and position (x-axis) of 5´ enriched clusters relative to the transcription start site (TSS). The distance is measured in base pairs from the TSS (TSS = 0). (E) Bar graph comparing the number (y-axis) and position (x-axis) of 5´ enriched clusters relative to the 5´-most exon-intron junction. The distance is measured in base pairs from the first exon-intron boundary.
(EPS)

S5 Fig Enriched cluster counts per gene and exon count of genes with enriched clusters. (A) The number of genes (y axis) plotted according to the specific number of enriched clusters (EC) per gene for both the high and low stringency datasets. Additional enriched cluster statistics are given in the legend. (B) The number of exons (x-axis) was analyzed for the genes identified as containing enriched clusters. The enriched clusters were further divided into several different categories. For reference, the average number of exons in the entire human genome is shown (red). The “genes in all the enriched clusters” category includes the annotated genes of all the enriched clusters identified by our analysis (yellow). Genes with clusters at 5´ end represents the annotated genes from clusters concentrated at the 5´ ends of transcripts (blue-green; Fig. 4C, bracket). The light green line (genes with clusters at 3´ end) represents the annotated genes from clusters concentrated at the 3´ ends of transcripts (0.8–1.0 in Fig. 4).
(EPS)

S6 Fig Steady-state levels of candidate transcripts after induction with dox. RNA was harvested at the indicated time points subsequent to lytic reactivation of TREx BCBL1-Rta cells with dox only (instead of butyrate and dox). (A) mRNA, (B) pre-mRNA, or (C) control RNA levels were monitored by RT-qPCR; positions of primers are shown in Fig. 5. We note that the kinetics and magnitude of changes in gene expression are reduced compared to the data in Fig. 6, but this is expected, as dox induction is less robust than butyrate and dox. Values were first normalized to 7SK RNA and the corresponding value for the 0 hpi time point was set to 1.0. 7SK RNA panel was only normalized to values from 0 hpi, but each experiment used equal amounts of total RNA. Mean values and standard deviation are shown (n = 3).
(EPS)

S7 Fig Selection of RT-induced mutations characteristic of ORF57 crosslinking. (A) The ratio of the total number of each type of mutation in the pellet tags divided by the number of mutations found in the input samples. All three biological replicates were combined into a single value for both the input and pellet mutation counts. (B) The total mutation count for each type of mutation for each sample in the sequencing data. The input samples (I1-I3) are displayed in shades of orange whereas the pellet samples (P1-P3) are shown in gray or black. The numbers refer to the three independent biological replicates. (C) The total mutation count in each of the three input samples was divided by the number of sequencing tags in the same samples to determine a mutation rate (orange). The same calculation was performed for the pellet samples (black). The numbers above the brackets are the fold differences between the pellet rates relative to the input rates for that mutation type. (D) Pearson correlations were determined comparing the total tag and mutant tag count from all bins in all clusters for each mutation type. A correlation for each of the six samples was determined as indicated.
(EPS)

S1 Table Enriched clusters in human and KSHV genomes. The coordinates for all enriched clusters in the human and viral genomes are listed for both the high and low stringency datasets. The first sheet provides a description of the data found in each of the columns. The additional spreadsheet contains the data.
(XLSX)

S2 Table GO Analysis of human enriched clusters. This spreadsheet includes GO analysis of genes from enriched clusters from both the high and low-stringency datasets. The first sheet provides a description of the nomenclature and data found on each sheet.
(XLSX)

S3 Table RT-qPCR primers. This table provides the names and sequences for all primers used in this study.
(DOCX)

S4 Table Mapping statistics. The number of reads sequenced and mapped for these studies are given for three independent biological replicates of the input and pellet samples. The distribution of mapped reads to the human (hg19 reference genome) or KSHV genomes (U75698.1) is shown.
(DOCX)

S5 Table Sites of characteristic mutations. The chromosomal location of each of the T→C or deletion mutations is shown as is the p-value that corresponds to the statistical test applied to determine whether the mutation was overrepresented in the pellets compared to the inputs (see Materials and Methods).
(XLSX)
