# Supplementary material for: HITS-CLIP Analysis Uncovers a Link between the Kaposi’s Sarcoma-Associated Herpesvirus ORF57 Protein and Host Pre-mRNA Metabolism
Source: PLoS Pathog. 2015 Feb 24;11(2):e1004652. doi: 10.1371/journal.ppat.1004652 (PMC4339584; doi:10.1371/journal.ppat.1004652)
Supplement: S3 Table — This table provides the names and sequences for all primers used in this study. (DOCX) [file ppat.1004652.s010.docx]

| **Supplementary Table S3. RT-qPCR primers** | | | |
| --- | --- | --- | --- |
| **Target** | **Direction** | **Primer name** | **Sequence (5'-3')** |
| BTG1 mRNA | Forward | NC1780 | GAGCTGCTGGCAGAACATTA |
| BTG1 mRNA | Reverse | NC1781 | GAACAGCTCCTGACTGCTCA |
| BTG1 pre-mRNA | Forward | NC1782 | GTCACCGGCACAATTAACAG |
| BTG1 pre-mRNA | Reverse | NC1783 | TGCACACAATGGAGTTGATG |
| TNFSF9 exon | Forward | NC1829 | GGCCTGAGCTACAAAGAGGA |
| TNFSF9 exon | Reverse | NC1830 | CCGCAGCTCTAGTTGAAAGA |
| TNFSF9 pre-mRNA | Forward | NC1845 | CACAAGCTCTGCATCTCTGG |
| TNFSF9 pre-mRNA | Reverse | NC1846 | AGGCTCTTTGGGAGTTAGCA |
| ZFP36 mRNA | Forward | NC1794 | ACTGCCATCTACGAGAGCCT |
| ZFP36 mRNA | Reverse | NC1795 | GACTCAGTCCCTCCATGGTC |
| ZFP36 pre-mRNA | Forward | NC1798 | GACTGCCATCTACGAGGTGA |
| ZFP36 pre-mRNA | Reverse | NC1799 | AGTTTGCGGCGCTAGAGA |
| EGR1 mRNA | Forward | NC1786 | CACCTGACCGCAGAGTCTT |
| EGR1 mRNA | Reverse | NC1787 | AAGCGGCCAGTATAGGTGAT |
| EGR1 pre-mRNA | Forward | NC1788 | GCGTCAGCTGTTGTTGAAAT |
| EGR1 pre-mRNA | Reverse | NC1789 | CTACCATTGACTCCCGAGGT |
| 7SK RNA | Forward | NC1164 | TAAGAGCTCGGATGTGAGGGCGATCTG |
| 7SK RNA | Reverse | NC1165 | CGAATTCGGAGCGGTGAGGGAGGAAG |
| GAPDH mRNA | Forward | NC638 | AGCCTCAAGATCATCAGCAATG |
| GAPDH mRNA | Reverse | NC639 | ATGGACTGTGGTCATGAGTCCTT |
| GAPDH pre-mRNA | Forward | NC1240 | TCCCCTCCTCATGCCTTCTT |
| GAPDH pre-mRNA | Reverse | NC1241 | CCAGGCGCCCAATACG |
| PAN RNA | Forward | NC702 | gctcgctgcttgccttctt |
| PAN RNA | Reverse | NC703 | ccaaaagcgacgcaatcaa |
| B-Actin mRNA | Forward | NC1230 | CTTCAACACCCCAGCCATGT |
| B-Actin mRNA | Reverse | NC1231 | CCAGAGGCGTACAGGGATAGC |
| B-Actin pre-mRNA | Forward | NC1821 | AGGGCTTCTTGTCCTTTCCT |
| B-Actin pre-mRNA | Reverse | NC1822 | CATAGGAATCCTTCTGACCCA |
| rRNA | Forward | NC1597 | AACCCGTTGAACCCCATT |
| rRNA | Reverse | NC1598 | CCATCCAATCGGTAGTAGCG |
