# Supplementary material for: HITS-CLIP Analysis Uncovers a Link between the Kaposi’s Sarcoma-Associated Herpesvirus ORF57 Protein and Host Pre-mRNA Metabolism
Source: PLoS Pathog. 2015 Feb 24;11(2):e1004652. doi: 10.1371/journal.ppat.1004652 (PMC4339584; doi:10.1371/journal.ppat.1004652)
Supplement: S4 Table — The number of reads sequenced and mapped for these studies are given for three independent biological replicates of the input and pellet samples. The distribution of mapped reads to the human (hg19 reference genome) or KSHV genomes (U75698.1) is shown. (DOCX) [file ppat.1004652.s011.docx]

| **Supplementary Table S4. Mapping Statistics** | | | | | | |
| --- | --- | --- | --- | --- | --- | --- |
| **Sample** | **Replicate** | **Total Reads** | **Mapped Reads (hg19)** | **Percent hg19** | **Mapped Reads**  **(KSHV)** | **Percent KSHV** |
| Input | 1 | 69047995 | 27933655 | 40.4% | 22887292 | 33.1% |
| Input | 2 | 97841141 | 41396437 | 42.3% | 35282358 | 36.0% |
| Input | 3 | 83944131 | 36403063 | 43.3% | 29800739 | 35.5% |
| Pellet | 1 | 60549062 | 12368578 | 20.4% | 1924779 | 3.17% |
| Pellet | 2 | 41225319 | 13582522 | 32.9% | 2241643 | 5.43% |
| Pellet | 3 | 56835116 | 8441660 | 14.8% | 1827394 | 3.21% |
